# The interplay among individuals’ distress, daily activities, and perceptions of COVID-19 and neighborhood cohesion: A study using network analysis

**DOI:** 10.1371/journal.pone.0293157

**Published:** 2024-01-18

**Authors:** Zhenchuan Yang, Jianwei Huang, Mei-Po Kwan, Dong Liu

**Affiliations:** 1 Institute of Space and Earth Information Science, The Chinese University of Hong Kong, Shatin, Hong Kong, China; 2 Department of Geography and Resource Management, The Chinese University of Hong Kong, Shatin, Hong Kong, China; 3 Institute of Future Cities, The Chinese University of Hong Kong, Shatin, Hong Kong, China; National Cheng Kung University College of Medicine, TAIWAN

## Abstract

The reduction of social interactions through non-pharmaceutical interventions (NPIs) has been shown to effectively curb COVID-19 transmission. However, these control measures were often accompanied by changes in people’s daily routines and constraints on their activity space, which could lead to mental distress (i.e., anxiety and depression). This study examined the interplay among individuals’ anxiety, depression, daily activities, and perceptions of COVID-19 and neighborhood cohesion. Taking Hong Kong as an example, an online survey (N = 376) was conducted to collect data from participants between March 14 to May 11, 2022. The data include respondents’ self-reported anxiety and depressive symptoms, daily activities (e.g., smartphone use), perceptions of COVID-19 (e.g., the possibility of infecting COVID-19), and perceptions of neighborhood cohesion. Using network analysis, we found that excessive smartphone use, life disturbance by COVID-19, and a community with people getting along well with each other were significant factors associated with participants’ anxiety and depression. Using critical path analysis, we observed that NPIs reduced human mobility, led to delayed bedtime, and increased smartphone use, which were associated with participants’ mental distress. We also found that NPIs and COVID-19 were associated with people’s perceptions of infection and the severity of COVID-19 and human mobility flexibility, which may further lead to mental distress. Our results also demonstrated that people with high education levels were vulnerable. These results provided important insights for designing appropriate interventions without generating deleterious impacts on people’s mental health in the future.

## Introduction

The COVID-19 pandemic is a serious global public health crisis. Countries around the world have resorted to vaccination and non-pharmaceutical interventions (NPIs) to mitigate the effect and control the transmission of the disease [1–3]. Before the mass vaccination, the reduction of people’s social interactions through NPIs effectively curbed COVID-19 transmission [1, 4]. However, these interventions were often accompanied by changes in people’s daily routines and constraints on their activity space, which could lead to mental distress (i.e., anxiety and depression) [5, 6]. For instance, it was reported that anxiety and major depressive distress increased by 25.6% and 27.6% globally in 2020 due to the COVID-19 pandemic and the associated control measures [7]. People’s suicidal thoughts and behaviors were also found to increase due to the mental distress caused by the pandemic [8]. Moreover, vulnerable populations—women, healthcare workers, younger people, people with pre-existing mental disorders, people with lower educational attainment, low-income people, and unemployed people—have been experiencing disproportionate COVID-19-related mental distress [9–12].

Apart from people’s sociodemographic attributes, previous studies have reported different factors associated with increased anxiety and depression during the early stages of the COVID-19 pandemic under strict control measures. Certain features of the natural and built environment have been found to have significant associations with people’s mental health [13, 14]. Notably, it was argued that such association might vary and should be interpreted with respect to specific contexts [15]. The control measures of governments were negatively associated with mental health, as observed in 15 Global North countries [16], and such association was mediated by observed physical distancing and perceptions of the government’s handling of the pandemic. A study [17] found that people in locked-down cities in Australia had poorer mental health. Besides, people’s daily activities that were affected by the control measures (e.g., physical exercises, sleep disturbances, and smartphone use) also had close relationships with their anxiety or depression. For example, increased physical exercise was associated with reduced depressive symptoms in Norway [9]. Previous studies [18, 19] observed that COVID-19 anxiety symptoms were related to excessive smartphone use in Chinese adults and children. It was also reported that people’s sleep quality and sleep habits were impaired due to their elevated mental distress in Italy during the pandemic [20].

The perceptions of COVID-19 risks (e.g., perceived uncontrollability or severity of COVID-19) were also reported to have positive associations with people’s mental distress. Evidence in 112 counties from [21] indicated that a higher perceived COVID-19 risk was associated with less positive (e.g., calm, content, and excited) or more negative (e.g., anxious, bored, and depressed) emotions. The “perceived uncontrollability” was also found to have a strong association with depression in China, and the association was moderated by social support (from family, friends, colleagues, and classmates) [22, 23]. In addition to the social support from family, friends, colleagues, and classmates, people’s perceptions of neighborhood cohesion were also important in affecting their mental health. For instance, a study observed that higher neighborhood cohesion was associated with lower odds of depression and anxiety in the U.S. [24]. A similar relationship between the perception of neighborhood and mental health was found in Beijing [25].

Previous studies have provided an important and fundamental understanding of the associations between individuals’ mental distress (i.e., anxiety and depression) and their daily activities, perceptions of COVID-19, and perceptions of neighborhood cohesion [26–28]. However, to the best of our knowledge, few studies simultaneously considered all these factors and presented a holistic picture of the interplay among individuals’ mental distress, daily activities, perceptions of COVID-19, and perceptions of neighborhood cohesion, which is considered crucial for formulating effective prevention and treatment strategies and measures. Moreover, traditional methods in previous studies—including descriptive analysis [29], regression models [16], structural equation models [18], and mediation models [30]—do not seem to adequately support such an interplay investigation.

In order to fill the abovementioned gap, this study applied network analysis to examine the interplay among individuals’ anxiety and depression and the determining factors in Hong Kong during the fifth wave of the COVID-19 pandemic. The psychological network analysis method was developed by psychologists for exploring the potential structure in which psychological and other components interplay with each other [31–33]. Based on the network analysis method, mental distress and the associated factors, including people’s daily activities, perceptions of COVID-19 risk, and perceptions of neighborhood cohesion were represented by nodes and connected by edges representing statistical associations in the estimated network.

## Materials and methods

**Fig 1:**
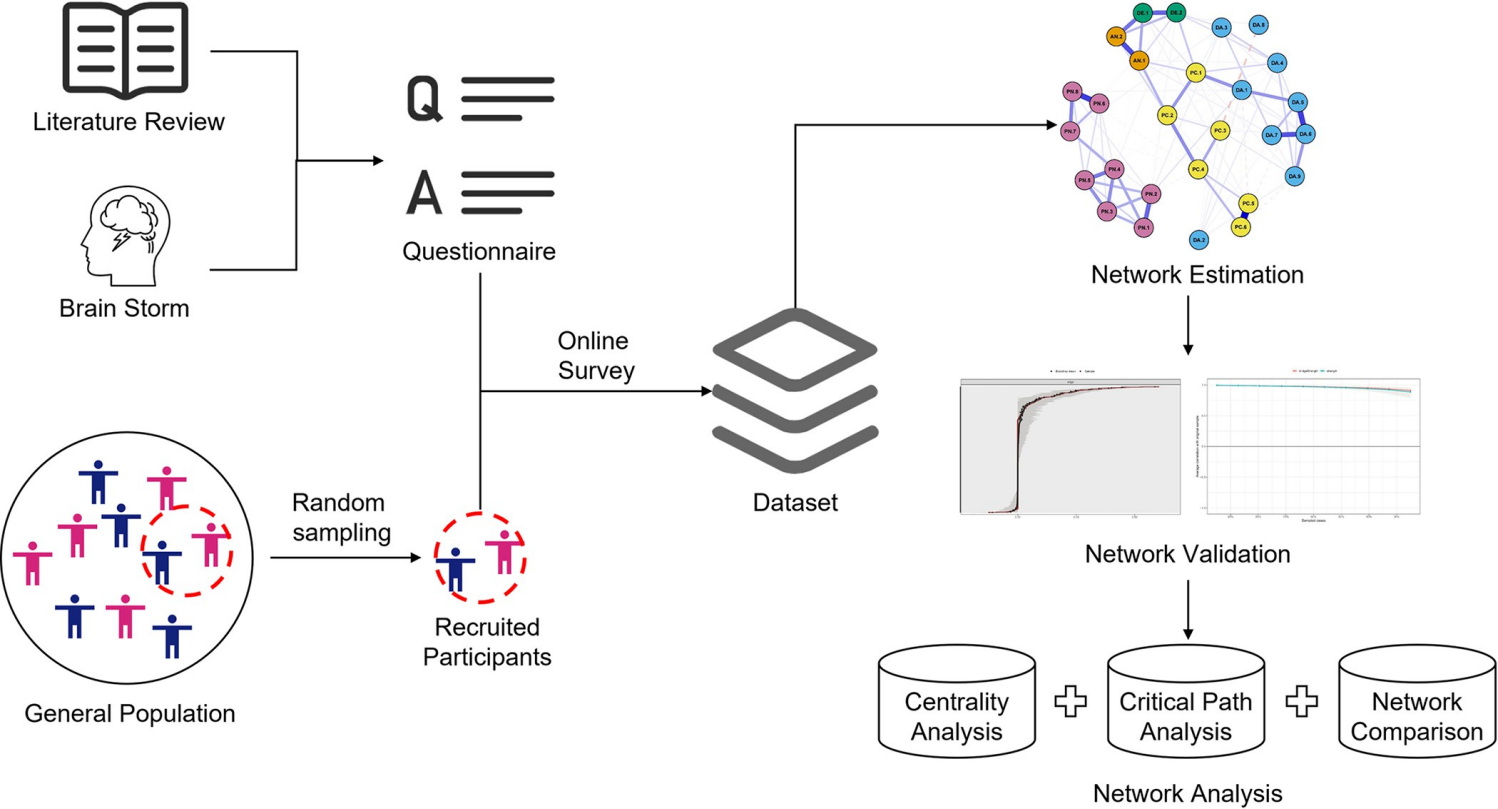
Research flow of our online cross-sectional survey and three procedures of network analysis.

In this study, we designed a research flow (see Fig 1) to address the abovementioned research gap. First, based on previous studies and brainstorming, we constructed a questionnaire to measure people’s mental distress, daily activities, perceptions of COVID-19 and perceptions of neighborhood cohesion. Second, we recruited participants from the general population of Hong Kong. Then, we conducted an online cross-sectional survey on the recruited participants to collect data. After that, this study applied the network analysis methods to investigate the collected data, which included three procedures: network estimation, network validation, and network analysis.

### Survey participants

Hong Kong was selected as the study area because it was hit severely by the COVID-19 pandemic before and during the present study’s period evidenced by the highest reported cases of 76,991 on 03/03/2022 and the highest deaths of 299 on 11/03/2022. And the government had imposed strict control measures to curb COVID-19 transmission, such as closing food services during the evening hours, closing entertainment venues, and suspension of mass events to avoid group gatherings. The online cross-sectional survey (N = 376) was conducted via the Qualtrics platform and recruited participants through several online channels (i.e., Facebook, Twitter, and Mass Email) from Mar 14, 2022 to May 11, 2022. The inclusion criteria of participants were the following: (1) Hong Kong residents living in Hong Kong during the fifth wave of COVID-19; (2) capable of reading and understanding traditional Chinese characters; (3) not infected with COVID-19 during the study period; (4) provided online electronic informed consent; (5) owned and able to use smartphones; (6) aged 18 years or older. The Survey and Behavioural Research Ethics Committee of the Chinese University of Hong Kong approved the study protocol. Informed consent was obtained from all participants through an electronic informed consent form prior to their inclusion in the study. To indicate their agreement to participate in the study, participants were required to click on an ‘Agree’ button within the electronic consent form. Eventually, 376 participants were recruited to participate in the survey. The distribution of participants based on sociodemographic characteristics (i.e., age, gender, marital status, education, income, employment, and residential location) was summarized in S1 Table in S1 File.

### Survey measures

The survey questionnaire has 27 questions in 5 categories: anxiety, depression, daily activities, perceptions of COVID-19, and perceptions of neighborhood cohesion. The details are shown in Table 1.

**Table 1:** Names and codes of the 27 response items in 5 categories in the survey questionnaire.

Categories	Items	Item codes
Anxiety	Feeling nervous, anxious, or on edge.	AN.1
	Not being able to stop or control worrying.	AN.2
Depression	Feeling down, depressed, or hopeless.	DE.1
	Little interest or pleasure in doing things.	DE.2
Daily activities	The influence of government restrictions on your daily activities.	DA.1
	Changes in 30 minutes or more of exercise over a week.	DA.2
	Changes in bedtime	DA.3
	Changes in smartphone use.	DA.4
	Changes in the frequency of your daily activities outside.	DA.5
	Changes in the frequency of going out.	DA.6
	Changes in the distance of going out.	DA.7
	To what extent can you decide on daily activities outside?	DA.8
	Changes in the time of staying home.	DA.9
Perceptions of COVID-19	Has COVID-19 affected your daily life?	PC.1
	How worried are you about COVID-19?	PC.2
	How likely are you to contract COVID-19 if not take any preventive measures?	PC.3
	How serious is the illness caused by contracting COVID-19?	PC.4
	Do you think there is a good chance that COVID-19 will be cured?	PC.5
	Do you think the chances of survival of COVID-19 patients are high?	PC.6
Perceptions of neighborhood	People in this community are willing to help their neighbors.	PN.1
	This is a close-knit community.	PN.2
	People in this community can be trusted.	PN.3
	People in this community get along well with each other.	PN.4
	People in this community can deal with problems together.	PN.5
	My neighbor and I share the same values.	PN.6
	I have good relations with my neighbors.	PN.7
	I share the same objective with my neighbors.	PN.8

#### Measuring anxiety symptoms (AN)

The Generalized Anxiety Disorder (GAD)-7 scale was often adopted to measure people’s anxiety symptoms, and its abbreviated version GAD-2 scale was evaluated to have good operating characteristics for detecting general anxiety as the GAD-7 [34, 35]. This study chose the well-established GAD-2 instrument as an effective and efficient screening tool [36], comprising two core anxiety symptoms (see Table 1). However, we transformed the traditional 4-point scale into a 6-point scale for each item to catch more information about the extent of people’s general anxiety. A high GAD-2 score represents a high level of anxiety.

#### Measuring depressive symptoms (DE)

The Patient Health Questionnaire (PHQ)-9 depression scale was extensively used in the literature to measure people’s depressive symptoms [34, 35]. And, as the brief version of PHQ-9, PHQ-2 becomes an attractive measure for depression screening due to its proven validity and effectiveness [37]. Therefore, this study used the PHQ-2 instrument, which includes 2 depressive symptoms (see Table 1). Likewise, we transformed the 4-point scale into a 6-point scale for each item in the PHQ-2. A high PHQ-2 score represents a high level of depression.

#### Measuring people’s daily activities (DA)

Smartphones have become central to our daily lives, and people’s daily activities can be divided into activities mediated by smartphone (ActMS) and activities not mediated by smartphone (ActNMS). Referring to [38], our study considered ActMS as people’s smartphone use, and people’s mobility, physical exercise, and sleep as three commonly measured ActNMS. And these activities have been found to be affected, to some extent, by COVID-19 and further influence people’s mental health [7, 18, 20, 39]. All items of people’s daily activities (see Table 1) were recorded on a 7-point Likert scale except the item “Changes in 30 minutes or more of exercise over a week” (DA.2), which measures people’s status of physical exercise and was recorded on a 11-point Likert scale. The category of people’s daily activities passed reliability and validity tests, and the results were presented in S2 Table in S1 File.

#### Measuring people’s perceptions of COVID-19 (PC)

Adjusted from the research of [29], this study divided people’s perceptions of COVID-19 into three aspects: overall worries, perceived susceptibility, and perceived severity, comprising six items (see Table 1). First, we asked about participants’ overall worry about COVID-19 and their daily life (i.e., PC.1 and PC.2). Then, we asked about participants’ perceived possibility of being infected with COVID-19 (i.e., PC.3). Finally, we asked about participants’ perceived severity of illness after being infected with COVID-19 (i.e., PC.4, PC.5, and PC.6). All items in the PC category were recorded on a 7-point Likert scale except PC.2, which measures the overall worry of COVID-19 for participants and was recorded on a 5-point Likert scale. The category of people’s perceptions of COVID-19 passed reliability and validity tests, and the results were presented in S2 Table in S1 File.

#### Measuring people’s perceptions of neighborhood cohesion (PN)

This study used established neighborhood cohesion questions, comprising 8 items that ask if people in the neighborhood are willing to help their neighbors, can be trusted, get along with one another, and share the same values, and whether this is a close-knit neighborhood [24, 40, 41]. All eight items were recorded on a 6-point Likert scale, and a high score represents perceived good neighborhood cohesion. The category of people’s perceptions of neighborhood cohesion passed reliability and validity tests, and the results were presented in S2 Table in S1 File.

#### Vulnerable subgroups

To evaluate the effect of COVID-19 on vulnerable people’s mental health, we divided participants into different subgroups based on their sociodemographic characteristics, including age, gender, education, income, marital status, employment, and residential location [42]. The details of the vulnerable subgroups are summarized in S1 Table in S1 File.

### Network analysis

The network analysis in this study consists of three procedures: (1) the estimation and visualization of the network, (2) the accuracy and stability validations of the estimated network, and (3) the analysis of the estimated network. All three procedures of the network analysis were conducted with RStudio version 2022.07.1–576 and the self-reported data of the 376 participants. Among them, the details of the second procedure (i.e., the accuracy and stability validations of the estimated network) were presented in S1 Text in S1 File.

#### The estimation and visualization of the network

In our study, the estimation of the network used the graphical least absolute shrinkage and selection operator (gLASSO) in combination with the extended Bayesian information criterion (EBIC) model selection (EBIC-gLASSO). The EBIC-gLASSO approach works well in dealing with the small sample problem and returns a sparse network, which contains fewer edges and is more interpretable [43, 44]. The estimation and visualization of the network were both conducted using the R package *bootnet* version 1.5 [45]. The estimated network consisted of nodes representing different items in Table 1, connected by edges representing unique associations between two items, which is measured by partial correlation coefficients. Thicker edges in the estimated network indicated stronger associations between the two items.

#### The analysis of the estimated network

The analysis of the estimated network consists of three parts: centrality analysis, critical path analysis, and network comparison. Centrality indices selected in this study were strength and bridge strength. Strength was defined as the sum of the absolute weights of the edge connecting a certain node to all other nodes [46]. Bridge strength was defined as the sum of the absolute value of all edges between node A and all nodes that were not in the same cluster as node A (hereafter Bridge Strength I) [47]. Here, we also investigated the modified bridge strength, which is defined as the sum of the absolute value of all edge weights between node A in the daily activities, perceptions of COVID-19, or perceptions of neighborhood cohesion and all nodes that are in anxiety and depression (hereafter Bridge Strength II). The centrality analysis was conducted using the R packages *qgraph* version 1.9.2 [48], *bootnet* version 1.5 [45], and *networktools* version 1.5.0 [47].

Critical path analysis aimed to discover the interplay among different items so that the potential causal relationships behind respondents’ mental distress may be highlighted and effective interventions can be identified. It originated from the field of project management. It seeks to identify tasks that are necessary and important for project completion [49]. Here, we conducted critical path analysis by first identifying an item (item-A) that is directly connected to an anxiety or depression symptom. Then we searched for another item directly connected to the identified item-A and determined an item (item-B) with the highest edge-weight with item-A. Third, the first and second steps were repeated. Finally, the critical path analysis stopped at item-X, which is considered the origin of the critical path.

Further, network comparison was conducted between subgroups using the R package *network comparison test* version 2.2.1 [50] based on age, gender, education, income, marital status, employment, and residential location. The network comparison consisted of three tests: i.e., network invariance tests, global strength invariance tests, and edge invariance tests. Based on the results of the network comparison, we detected the impact of social determinants on the interplay among anxiety, depression, daily activities, perceptions of COVID-19, and perceptions of the neighborhood.

## Results

### Descriptive analysis

**Fig 2:**
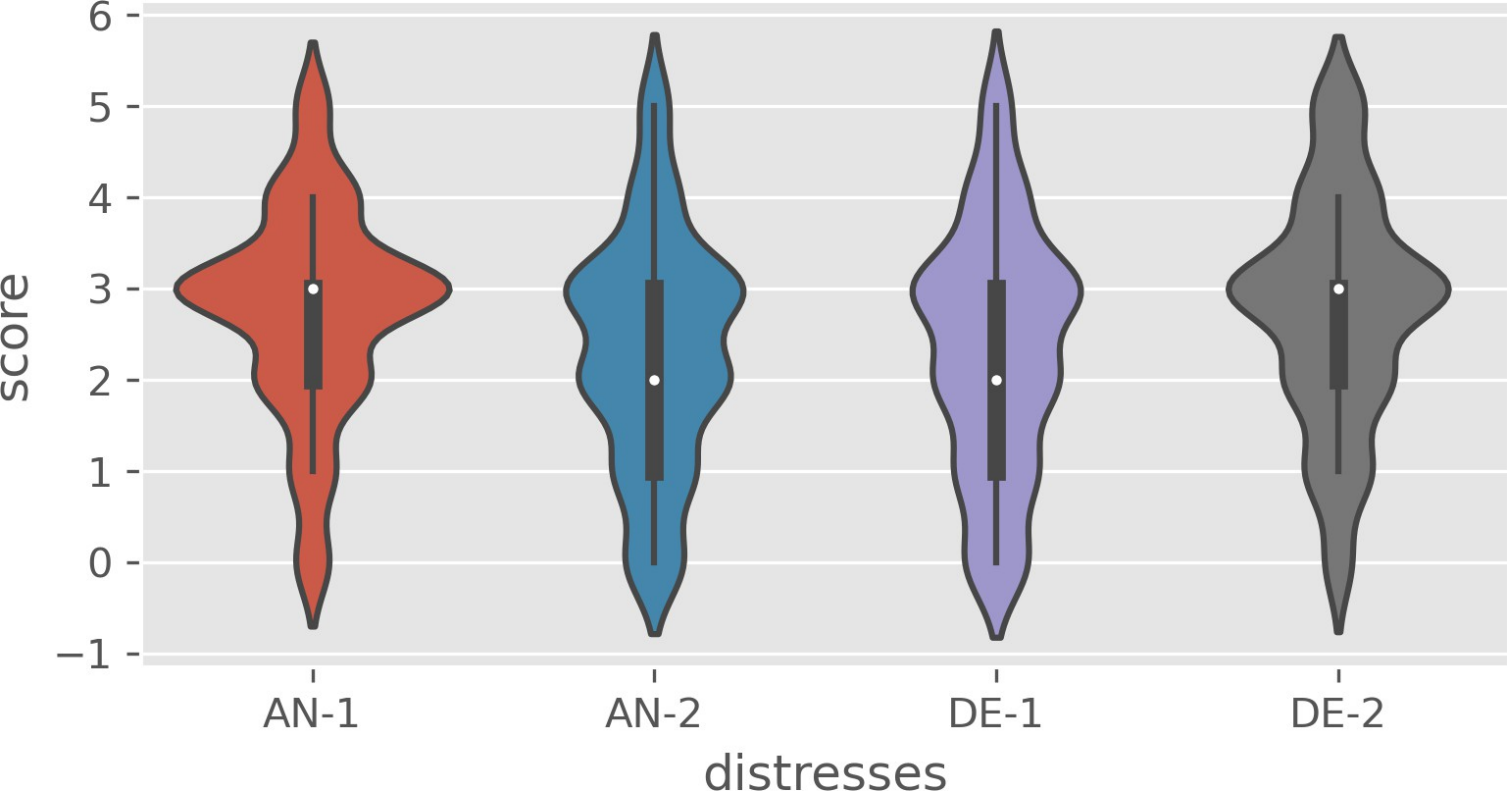
The distribution of four items depicting individuals’ anxiety and depression: Feeling nervous, anxious, or on edge (AN-1); Not being able to stop or control worrying (AN-2); Feeling down, depressed, or hopeless (DE-1); Little interest or pleasure in doing things (DE-2).

The distributions of the four anxiety and depression symptoms among all 376 participants were shown in Fig 2. The numbers on the vertical axis represent the extent to which respondents had specific anxiety or depression symptoms in the past 2 weeks: 0 is never; 1 is almost none, 2 is very few, 3 is sometimes, 4 is often, 5 is always. The height of the boundary represents the probability of each frequency. Fig 2 indicated that participants had a high frequency of “feeling nervous, anxious, or on edge” and “perceiving little interest or pleasure in doing things”. On the contrary, we discovered a low frequency of “not being able to stop or control worrying” and “feeling down, depressed, or hopeless”. More information about anxiety and depression symptom distribution based on age, gender, education, income, marital status, employment, and residential location can be found in S2 Text in S1 File.

### Estimated network

**Fig 3:**
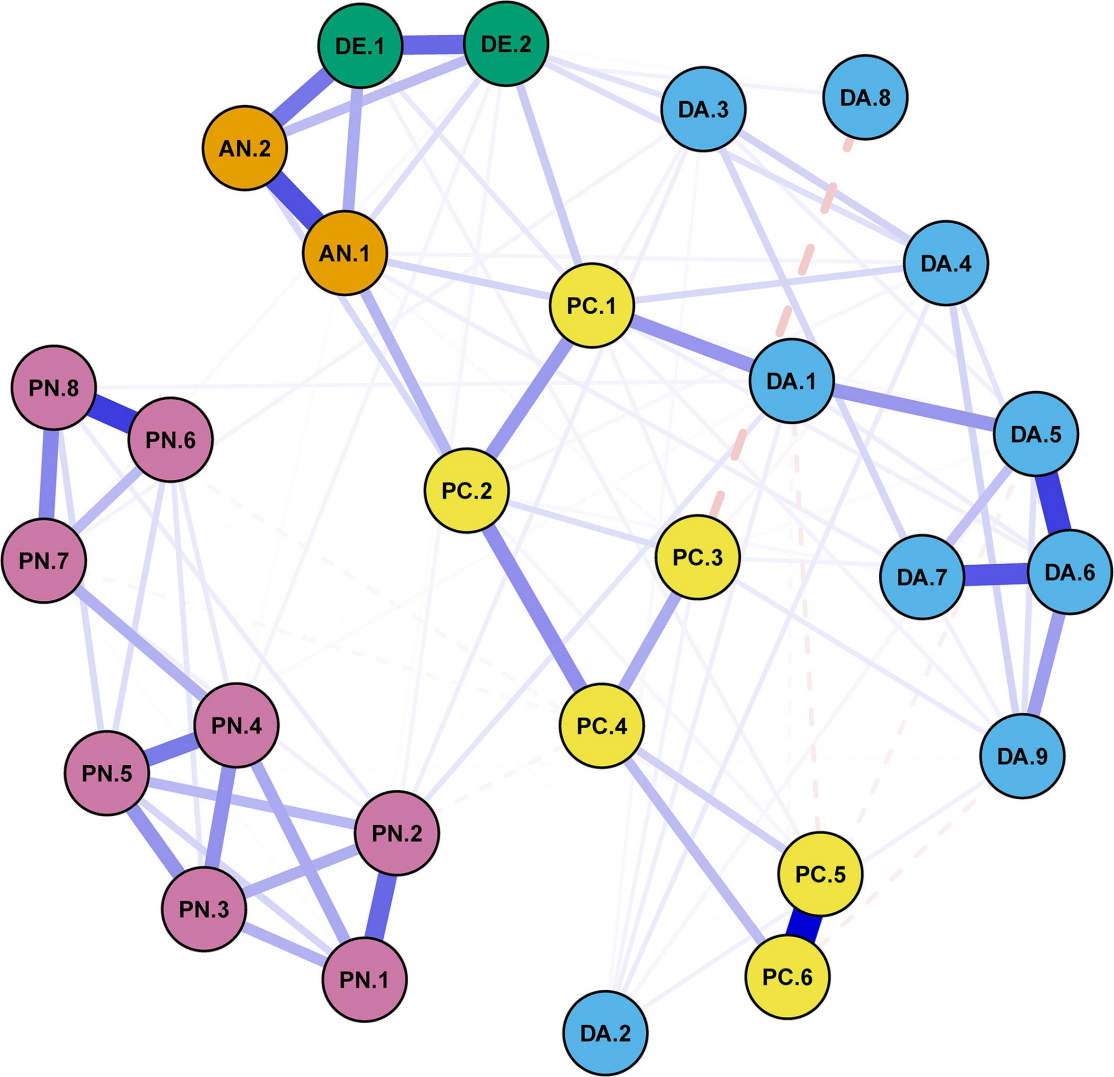
The estimated network of 27 items among anxiety (AN, in orange), depression (DE, in green), daily activities (DA, in light blue), perceptions of COVID-19 (PC, in yellow), and perceptions of neighborhood (PN, in purple).

The estimated network between the 27 response items was visualized in Fig 3, which depicted the interplay among participants’ anxiety, depression, daily activities, perceptions of COVID-19, and perceptions of neighborhood. In the network, the nodes represent the response items, and the edges represent the associations between the response items. Thicker solid and deeper blue edges mean higher positive associations, while thicker dashes and deeper orange edges represent higher negative associations. The values of association for each edge (i.e., edge-weight) were presented in S3 Table in S1 File. The estimated network was sparse due to the regulation of the EBIC-gLASSO approach and only had 104 non-zero edges out of 351 possible edges (i.e., the network density is 0.296). Fig 3 showed that stronger associations existed between items within each category, while weaker associations occurred between items from different categories. Positive associations were predominant in the network, while sparse negative associations also existed. For example, a strong negative association existed between “To what extent can you decide your daily activity outside” (DA.8) and “How likely are you to contract COVID-19 without taking any preventive measure?” (PC.3). This may be because respondents considered more freedom in making decisions about their daily activities outside will bring higher COVID-19 risk. The results of the network validation were presented in S3 Text in S1 File.

### Centrality indices

**Fig 4:**
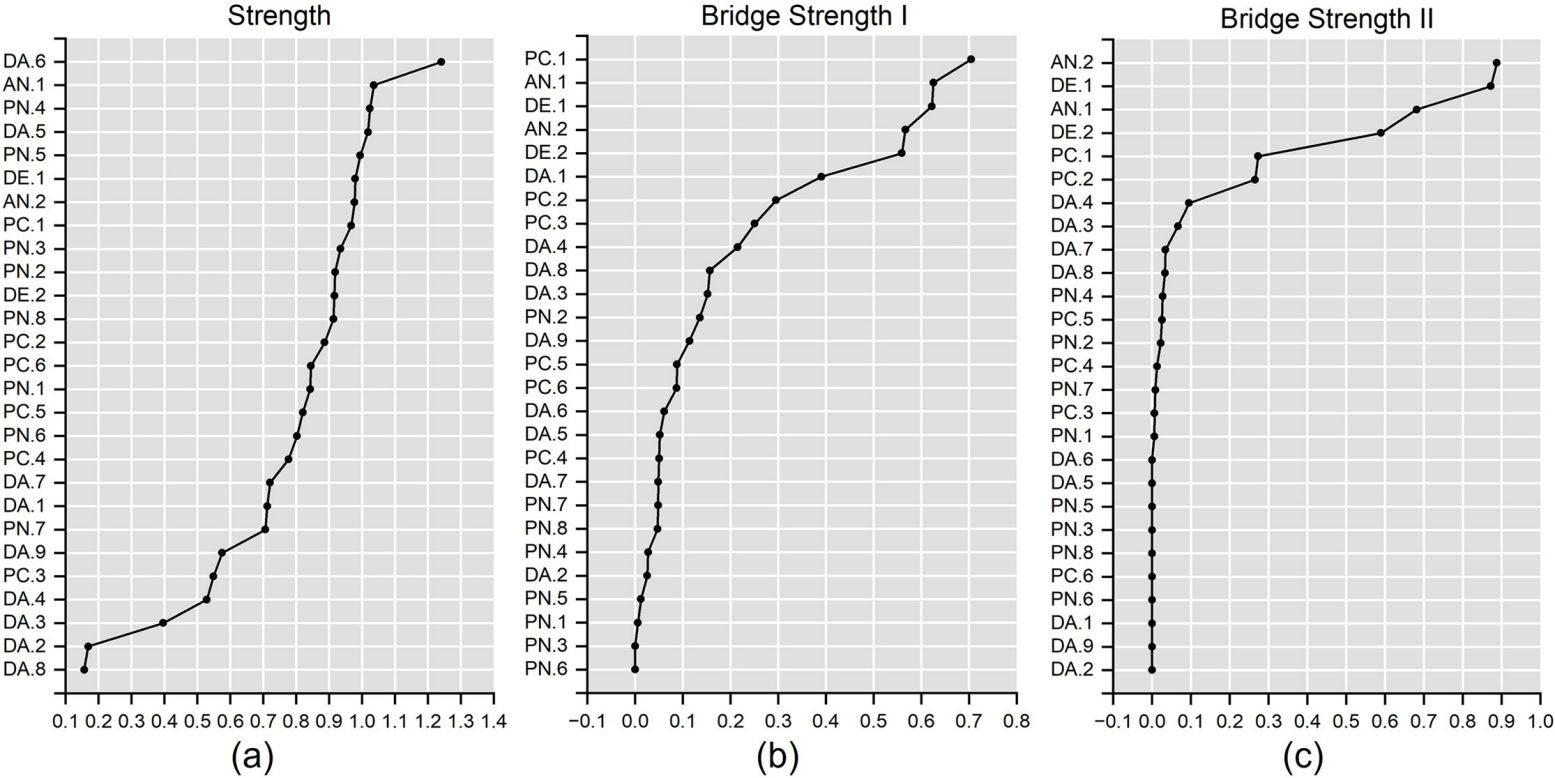
Strength (a), Bridge Strength I (b), and Bridge Strength II (c) of 27 items in descending order of their original values.

The centrality indices, including Strength, Bridge Strength I, Bridge Strength II were calculated and visualized as shown in Fig 4. The original values of Strength, Bridge Strength I, Bridge Strength II, and the descriptive statistics of each item were presented in S4 Table in S1 File. Fig 4(A) indicated that the Strength values of the response items differed quite substantially. And “Changes in the frequency of going out” (DA.6) had the highest Strength value, which was considered the most important item in the estimated network. Fig 4(B) showed that the values of Bridge Strength I of 27 items also differed significantly. The “Has COVID-19 affected your daily life?” (PC.1) in the perceptions of COVID-19 had the highest Bridge Strength I value, meaning that it had the strongest association with the items in anxiety, depression, daily activities, and perceptions of neighborhood. Fig 4(C) portrayed the results of Bridge Strength II, and we found that “Has COVID-19 affected your daily life?” (PC.1) had the highest value of Bridge Strength II as well, which revealed it had the highest associations with anxiety and depressive symptoms.

### Critical paths in the estimated network

Based on the critical path analysis described in the network analysis subsection and using the visualized network (Fig 3) and the values of associations for each edge (S3 Table in S1 File), we identified nine critical paths in the estimated network (as shown in Table 2). All critical paths started with an anxiety or depressive symptom. In this study, we assumed that the last item is “The influence of government’s restrictions on your daily activities” (DA.1) because the NPIs implemented by governments to control COVID-19 resulted in a chain reaction and eventually caused mental distress [17, 51, 52]. We discussed the nine critical paths in the conclusion and discussion section below.

**Table 2:** Nine identified critical paths in the estimated network (Fig 3).

No.	First item	Second item	Critical path
1	AN.1	PC.1	AN.1—PC.1—DA.1
2	AN.1 & AN.2	PC.2	AN.1 & AN.2—PC.2—PC.1—DA.1
3	DE.1	PC.5	DE.1—PC.5—PC.6—PC.4—PC.2—PC.1—DA.1
4	DE.1 & DE.2	PC.1	DE.1 & DE.2—PC.1—DA.1
5	AN.1	DA.7	AN.1—DA.7—DA.6—DA.5—DA.1
6	AN.1	DA.4	AN.1—DA.4—DA.3—DA.7—DA.6—DA.5—DA.1
7	DE.2	DA.3	DE.2—DA.3—DA.7—DA.6—DA.5—DA.1
8	DE.2	DA.4	DE.2—DA.4—DA.3—DA.7—DA.6—DA.5—DA.1
9	DE.1 & DE.2	DA.8	DE.1 & DE.2—DA.8—PC.3—PC.4—PC.2—PC.1—DA.1

### Network comparison based on subgroups

The results of the network invariance tests and global strength invariance tests were shown in S4 Fig in S1 File, which revealed that the network structures and global strengths were similar between subgroups of age, gender, income, marital status, employment, and residential location, while the network structures were distinct with respect to education. The results of the edge invariance test regarding the subgroups of education were shown in S5 Table in S1 File, which indicated that 10 edges (e.g., edge between PC.5 and PC.6) are significantly different between people with a high level of education and a low level of education. All estimated networks for subgroups based on age, gender, education, income, marital status, employment, and residential location were presented in S5 Fig in S1 File. We discussed the network comparison result in the conclusion and discussion section.

## Conclusion and discussion

### Centrality analysis

In our study, the centrality indicator of Bridge Strength I was primarily used for the network validation. And we concentrated on the Strength and Bridge Strength II in participants’ daily activities, perceptions of COVID-19, and perceptions of neighborhood cohesion to interpret the network.

In terms of daily activities, we estimated that “Changes in the frequency of going out” (DA.6) had the highest Strength (1.241) and “Changes in the use of smartphone” (DA.4) had the highest Bridge Strength II (0.096) according to Fig 4 and S4 Table in S1 File. Fig 3 further presented that the high Strength of DA.6 is due to its high internal associations with “Changes in the frequency of your daily activities outside” (DA.5) and “Changes in the distance of going out” (DA.7). The high within-category associations may be because DA.5, DA.6, and DA.7 are different aspects of human mobility. We also found that “Changes in the use of smartphone” has the strongest association with anxiety and depression among items in respondents’ daily activities. This result was in line with previous studies, which observed an interplay between people’s anxious/depressive symptoms and smartphone use: anxiety and depression prompt people to seek relief by increasing smartphone use, while excessive smartphone use inversely aggravates people’s anxiety and depression [18].

Regarding the items in perceptions of COVID-19, Fig 4 and S4 Table in S1 File revealed that “Has COVID-19 affected your daily life?” (PC.1) had both the highest values of Strength (0.967) and Bridge Strength II (0.273). This result showed that people’s perception of daily life disturbed by COVID-19 had the strongest and broadest associations with other items in all categories. It should be noted that previous studies also concluded that the disruption of people’s daily life and work, which was induced by mobility restrictions of COVID-19 control measures, may give rise to anxiety and depression [53].

In terms of the items in perceptions of neighborhood, Fig 4 and S4 Table in S1 File indicated that “People in this community get along well with each other” (PN.4) had both the highest Strength (1.024) and Bridge Strength II (0.027). The high Strength of PN.4 may be because of high within-category associations among the items in the perceptions of neighborhood, which revealed that a community with people getting along well with each other, is the key factor that influences neighborhood cohesion. Although PN.4 also had the highest Bridge Strength II, it is relatively low compared to “Changes in smartphone use” and “Has COVID-19 affected your daily life?” items. This result may be because of the sparse and weak associations between PN.4 and anxious and depressive symptoms.

### Critical path analysis

Based on the estimated network (Fig 3), we found that some items in the perceptions of neighborhood (e.g., PN.4: “People in this community get along well with each other”) are directly connected to anxiety or depression symptoms. The positive associations of such connections revealed that a decrease in neighborhood cohesion might slightly raise depression and anxiety, which [24] echoed. Nonetheless, the Bridge Strength II values of the items in the perception of neighborhood are much weaker when compared with that in the items of perceptions of COVID-19 and daily activities. In other words, our results implied that the loss of neighborhood cohesion might not generate severe anxiety and depression symptoms in Hong Kong during the fifth wave of the COVID-19 outbreak.

Based on critical paths 1, 2, 3, 4, and 9 in Table 2, we identified one basic path: “DA.4—DA.3—DA.7—DA.6—DA.5—DA.1”. This basic path consisted of two components of daily activities: the changes in indoor activities and human mobility. The sub-path “DA.7—DA.6—DA.5—DA.1” represents the interplay between human mobility and government restrictions. In the case of Hong Kong, the government implemented strict social distancing measures during the fifth wave of the COVID-19 outbreak in the city, such as closing night food service, closing entertainment venues, and suspending mass events to avoid group gatherings (DA.1). These social distancing measures reduced people’s mobility, including the frequency of outdoor activities, the frequency of travel, and travel distance (i.e., DA.5, DA.6, and DA.7), which was echoed in [7]. On the other hand, “DA.4—DA.3” portrayed the interplay between indoor activities (i.e., the changes in bedtime (DA.3) and smartphone use (DA.4)) induced by the change in human mobility. The DA.3 may be because the decreased human mobility (DA.5, DA.6, and DA.7), especially working from home, dissolved the boundaries between weekdays and weekends as well as days and nights. As a result, people’s work was interspersed between relaxations (e.g., watching television) and frequent naps, which influenced people’s regular bedtime [53, 54]. We also discovered a positive association between changes in bedtime (DA.3) and smartphone use (DA.4). This interplay can be interpreted as excessive smartphone use leading to sleep impairment such as delayed bedtime [55]. And the sleep impairment was aggravated by even more smartphone use when people cannot fall asleep [56, 57].

Based on the basic path of “DA.4—DA.3—DA.7—DA.6—DA.5—DA.1”, we further interpreted how it can connect to anxiety and depressive symptoms. Critical path 5 in Table 2 showed that the changes in travel distance (DA.7) were directly associated with anxiety symptoms (AN.1), which might be because Hong Kong residents are more sensitive about the change in travel distance when compared with the frequencies of outdoor activities (DA.5) and travels (DA.6). Based on critical paths 6 and 8 in Table 2, we detected direct associations between the increase in smartphone use (DA.4) and anxiety (AN.1) and depressive symptoms (DE.2). A possible reason is that people were more frequently exposed to COVID-19 related news, which increase anxiety and depression [58, 59]. On the contrary, people tended to seek relief from anxiety and depression on smartphones, such as entertainment from movies and games, which were easily accessible means under the strict social distancing measures [18, 60]. Finally, we also found a positive association between delayed bedtime (DA.3) and the rise of depressive symptoms (DE.2). Similar evidence has been observed in references [61, 62]. Remarkably, delayed bedtime may increase anxiety as observed in existing literature [55, 63]. However, the current study cannot find such a correlation in Hong Kong during the fifth wave of the COVID-19 outbreak.

Based on critical paths 5, 6, 7, and 8 in Table 2, we extracted another basic path: “PC.4—PC.2—PC.1—DA.1”. In terms of the basic path of “PC.4—PC.2—PC.1—DA.1”, the positive association between PC.1 and DA.1 indicated that participants’ daily life and activities were both affected by the perceptions of COVID-19 (PC.1) and government restrictions (DA.1). On the one hand, participants’ daily life and activities (e.g., working from home, delayed bedtime, and excessive smartphone use) might be affected by government interventions (e.g., social distancing measures) [29, 64–66]. On the other hand, the change in participants’ daily lives driven by COVID-19 might be because individuals feared the severity after infecting COVID-19 and took self-protective behaviors (e.g., avoiding going to crowded places). This interplay was also reflected as the path “PC.4—PC.2—PC.1”.

Based on the basic path of “PC.4—PC.2—PC.1—DA.1”, we further interpreted how it is connected to anxious and depressive symptoms. Critical paths 1 and 4 (see Table 2) indicated that “Has COVID-19 affected your daily life?” (PC.1) was detected to be directly associated with both anxious and depressive symptoms, which implies that the disturbance of daily life may directly increase people’s anxiety and depression [24, 30, 67, 68]. We also found that “How worried are you about COVID-19?” (PC.2) is directly connected to anxiety symptoms (see critical path 2 in Table 2). In fact, PC.2 can be decomposed into the perceived possibility of infecting COVID-19 (PC.3), and the perceived severity of illness after infecting COVID-19 (PC.4), and PC.4 can be further decomposed into the perceived curability chance after infecting COVID-19 (PC.5) and the survival chance after infecting COVID-19 (PC.6), which were verified by the strong association between these items in Fig 3 and critical paths 3 and 9. And previous studies also indicated that these perceptions of COVID-19 had high and positive associations with people’s anxiety or depression [21, 29, 69, 70]. Our study also found a negative association between “To what extent you can decide daily activity outside.” (DA.8) and PC.3 in critical path 9. The possible interpretation was that people’s worries about infecting COVID-19 (PC.3) restrict the extent to which they can freely decide on their daily outdoor activities (DA.8). Finally, this study discovered a positive association between DA.8 and depressive symptoms (DE.1 & DE.2) in critical path 9. This result was also observed by [71], which indicated that the loss of mobility flexibility would increase depression and even cause suicides during the COVID-19 pandemic.

### Network comparison

**Fig 5:**
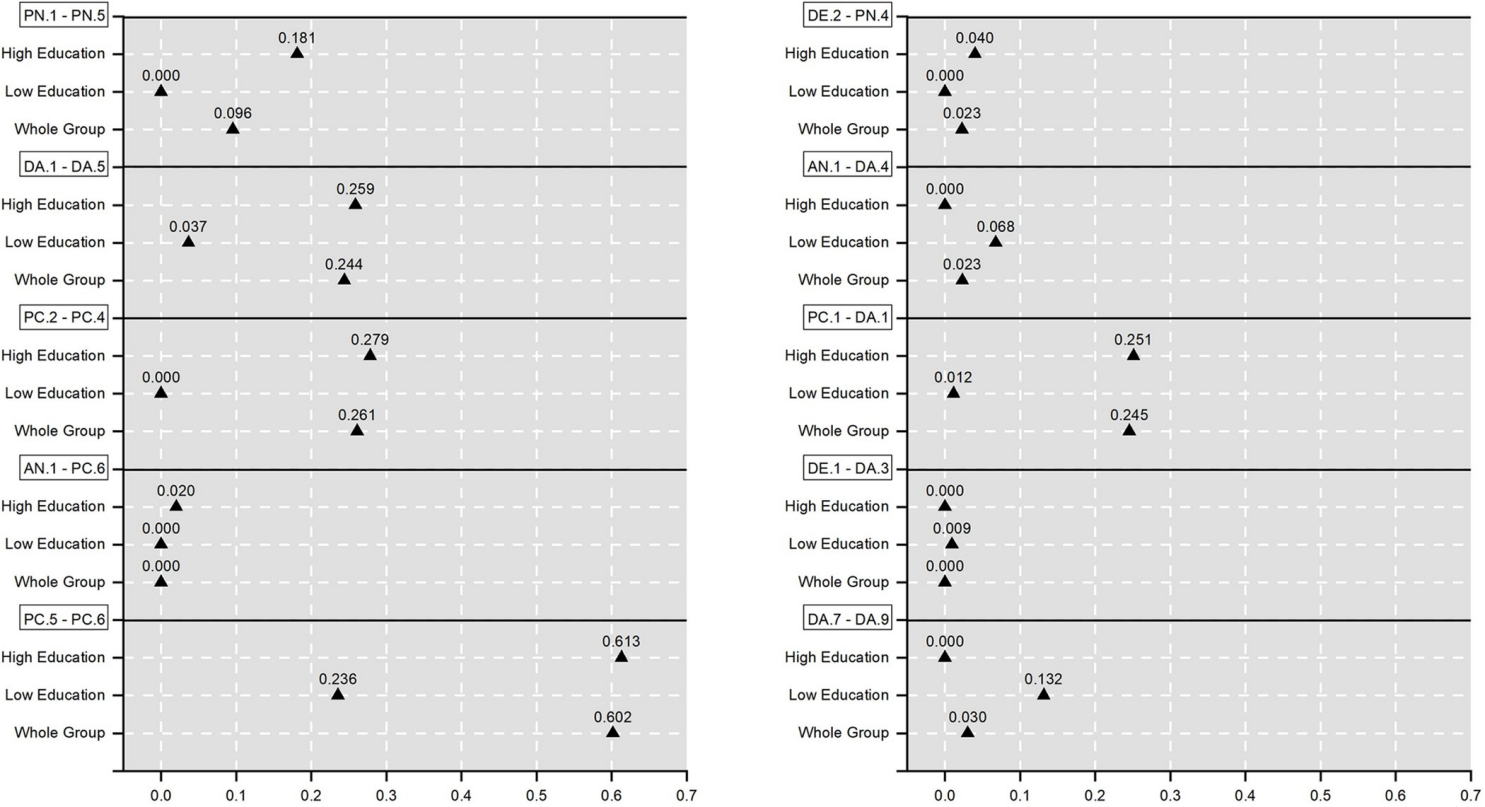
Edge-weights of ten edges in the high education group, low education group, and whole group, respectively.

Network comparison was conducted between subgroups of age, gender, education, income, marital status, employment, and residential location, and we found that there are significant differences between the estimated networks between different subgroups of education level. Specifically, participants were divided into two subgroups, i.e., a subgroup with a high education level (Master’s degree or above, Post-secondary education: Degree programs, or Post-secondary education: Sub-degree programs) and a subgroup with a low education level (Post-secondary education: Diploma/Certificate, Secondary school education or equivalent, Primary school and below, or no formal education). Based on S5 Table in S1 File, we discovered ten edges in the estimated networks are significantly different in statistics. For better result illustration, we further transformed the result in S5 Table in S1 File into Fig 5. Moreover, we conducted a statistical analysis on participants’ self-reported anxiety and depressive symptoms between the high-education subgroup and the low- education subgroup, and the statistical result is shown in S6 Table in S1 File. Based on the mean values in S6 Table in S1 File, we discovered that, in our case, people in the high-education subgroup experienced more serious anxiety and depressive symptoms than people in the low education subgroup. This finding is in line with previous studies which found that higher education levels were associated with greater concerns about the consequences of COVID-19 (e.g., becoming seriously ill) because people with higher education levels may have greater engagement and interest in health information [72–74]. The sensitivity analysis of anxiety and depressive symptoms regarding participants with different settings of education levels was described in S4 Text in S1 File and the results were shown in S7 Table in S1 File.

Based on Fig 5, we found two sorts of edges, i.e., the edge-weights in the high education subgroup are stronger than that in the low education subgroup (including PN.1-PN.5, DA.1-DA.5, PC.2-PC.4, AN.1-PC.6, PC.5-PC.6, DE.2-PN.4, and PC.1-DA.1); and the edge-weights in the high education subgroup are weaker than that in the low education subgroup (including AN.1-DA.4, DE.1-DA.3, and DA.7-DA.9). Comparing to people with low-education levels, people with high-education levels had a stronger sense of neighborhood cohesion (PN.1-PN.5); were more influenced by government restrictions (DA.1-DA.5); were more worried about seriousness of the illness caused by COVID-19 (PC.2-PC.4); their anxious symptom of “feeling nervous, anxious, or on edge” was more likely to be affected by the perception of survival possibility of COVID-19 (AN.1-PC.6); were more sensitive about the relationship between perceived curability and survival due to COVID-19 (PC.5-PC.6); their depressive symptom of “Little interest or pleasure in doing things” was more likely to be influenced by the relationship between neighborhoods (DE.2-PN.4); and they had a stronger perception that their daily life and activities are disrupted by COVID-19 (PC.1-DA.1). On the other hand, in regard to people with low-education levels, their anxiety symptom of “feeling nervous, anxious, or on edge” was more like to be affected by smartphone use (AN.1-DA.4); their depressive symptom of “Feeling down, depressed, or hopeless” had a stronger association with changes in bedtime (DE.1-DA.3); and their travel distance was more sensitive to their time staying home (DA.7-DA.9).

### Contributions and limitations

To the best of our knowledge, our study is the first study applying network analysis to investigate the holistic interplay among individuals’ mental distress (i.e., anxiety and depressive symptoms) and various attributes of people’s daily activities, perception of COVID-19 risks, and perception of neighborhood cohesion under the context of COVID-19 pandemic. Traditional tools in network analysis, including centrality analysis and network comparison, were used in our study. On the one hand, we estimated a network of anxiety, depressive symptoms, and their associated factors and identified the key factors associated with respondents’ anxiety or depressive symptoms (i.e., smartphone use, people’s perception of daily life disturbed by COVID-19, and people’s perception of whether they can get along well with neighbors). Besides, we also found that the interplay between items in the estimated network is different between the high-education and low-education subgroups, and the people with high education levels are the vulnerable population.

Especially, this paper integrated critical path analysis from project management into network analysis. Referring to the critical path analysis, we identified nine critical paths. For example, we observed that NPIs tended to reduce human mobility, led to delayed bedtime, and increased smartphone use, which were associated with mental distress. We also found that NPIs and COVID were associated with people’s perceptions of infection and severity of COVID-19 and human mobility flexibility, which may further led to mental distress. Practically, the results from this study can be an important reference for governments to formulate tailor-made policies to facilitate post-pandemic recovery of people’s mental health for Hong Kong residents. The proposed methods are applicable to other cities around the world for developing future cities in a more equal and resilient manner that can better cope with future pandemic-induced mental health challenges.

However, this study also has limitations. First of all, due to the strict public health measures during the time of the survey, the sample size in our study is relatively small, which may affect its representativeness of the general population in Hong Kong. Second, this study is a cross-sectional study, which is weak in interpreting the causal relationships between different factors compared with cohort or longitudinal studies [75, 76]. Finally, due to data availability, this study did not consider the impact of the real-time natural and built environment and real-life infection rate on people’s anxiety and depression during the COVID-19 pandemic [77–79].

## Supporting information

S1 File Supplementary information for the interplay among individuals’ distress, daily activities, and perceptions of COVID-19 and neighborhood cohesion: A study using network analysis. (DOCX)
