# Supplementary material for: The interplay among individuals’ distress, daily activities, and perceptions of COVID-19 and neighborhood cohesion: A study using network analysis
Source: PLoS One. 2024 Jan 18;19(1):e0293157. doi: 10.1371/journal.pone.0293157 (PMC10796027; doi:10.1371/journal.pone.0293157)
Supplement: S1 File — (DOCX) [file pone.0293157.s001.docx]

**Supplementary information for**

**The interplay among individuals’ distress, daily activities, and perceptions of COVID-19 and neighborhood cohesion: A study using network analysis**

**This supplementary information includes:**

Text S1. The accuracy and stability validations of the estimated network using the EBIC-gLASSO approach.

Text S2. Descriptive analysis of four anxiety and depression symptoms among 376 participants.

Text S3. The results of accuracy and stability of estimated network using the EBIC-gLASSO approach.

Text S4. The sensitivity analysis of anxiety and depressive symptoms regarding participants with different education levels.

Table S1. Vulnerable subgroup setting of age, gender, education, income, marital status, employment, and residential location.

Table S2. The results of reliability and validity tests of questionnaires for people’s daily activities, people’s perceptions of COVID-19, and people’s perceptions of neighborhood cohesion.

Table S3. The weighted adjacency matrix storing the values of associations for each edge.

Table S4. Original values of Strength, Bridge Strength I, and Bridge Strength II, and the descriptive statistics of each item in the estimated network.

Table S5. The results of edge invariance test based on education. Here, the presented edges are different at a significant level of 0.053 (i.e., p-value = 0.053).

Table S6. The statistics of self-reported anxiety and depressive symptoms between the high education group and the low education group.

Table S7. The mean scores of anxiety and depressive symptoms between the high education group and the low education group under six different subgroup settings.

Fig S1. The distributions of four anxiety and depression items for different subgroups based on age (a), gender (b), education (c), income (d), marital status (e), employment (f), and residential location (g). Subfig (h) describes the distributions of four anxiety and depression items for the whole sample.

Fig S2. Bootstrapped confidence intervals (CIs) of estimated edge-weights for the estimated network.

Fig S3. Average correlations between strength and Bridge Strength I of networks sampled with case-dropped samples and the original sample.

Fig S4. The results of network invariance tests and global strength invariance tests based on (a) age, (b) gender, (c) education, (d) income, (e) marital status, (f) employment, and (g) residential location.

Fig S5. Estimated networks for subgroups based on (a) age, (b) gender, (c) education, (d) income, (e) marital status, (f) employment, and (e) residential location

**Text S1. The accuracy and stability validations of the estimated network using the EBIC-gLASSO approach.**

As suggested by [1], this paper conducted three kinds of validations for the estimated network, including edge-weight accuracy, centrality stability, and the testing for significant differences. The edge-weight accuracy was validated by assessing the 95% confidence intervals (CI) of edge-weights using a non-parametric 1000-time bootstrapping method [1, 2]. The 95% CI represents that in 95% of the cases, such a CI will contain the true value of the parameter. And when the bootstrapped CIs are narrow, it becomes easy to interpret the order of edges. The centrality stability was examined via correlation stability coefficients (CS-C) using a case-dropping 1000-time bootstrapping method [3]. The CS-C (cor = 0.7) represents the maximum proportion of cases that can be dropped, such that with 95% probability, the correlation between original centrality indices and centrality indices of networks based on subsets is 0.7 or higher [4]. Previous studies showed that the CS-C should not be below 0.25 and preferably above 0.5 [1]. In addition, the testing for significant differences between specific edge-weights and centrality indices of nodes was conducted using the bootstrapped difference test based on the bootstrapped results in validating edge-weight accuracy [5]. The above three validations were all performed using R package *bootnet* version 1.5.

**Text S2. Descriptive analysis of four anxiety and depression symptoms among 376 participants.**

The distributions of the four anxiety and depression symptoms among all 376 participants were shown in S1 Fig (h). S1 Fig (h) indicated that participants had a high frequency of “feeling nervous, anxious, or on edge” and “perceiving little interest or pleasure in doing things”. On the contrary, we discovered a low frequency of “not being able to stop or control worrying” and “feeling down, depressed, or hopeless”.

Moreover, we portrayed the distributions of the four anxiety and depression items for different subgroups based on age, gender, education, income, marital status, employment, and residential location, as shown in S1 Fig (a)-(f). Based on S1 Fig (a)-(f), we found that there was almost no difference in the frequency of “feeling nervous, anxious, or on edge” and “little interest or pleasure in doing things” in subgroups across all sociodemographic characteristics. On the contrary, differences in the frequency of “feeling down, depressed, or hopeless” appeared in subgroups of all sociodemographic characteristics. The young, females, people with a high level of education, people with low income, unmarried people, unemployed people, and people residing in the New Territories may be vulnerable populations because they had higher frequency of feeling down, depressed, or hopeless. In regard to “not being able to stop or control worrying”, we discovered females, people with low income, and unemployed people might be vulnerable populations.

We further conducted a statistical analysis on participants’ self-reported anxiety and depressive symptoms between the high education subgroup and the low education subgroup, and the statistical result is shown in S5 Table. Based on the mean values in Supplementary S5 Table, we discovered that, in our case, people in the high education subgroup experienced more serious anxiety and depressive symptoms than people in the low education subgroup. This finding is in line with previous studies which found that higher education levels were associated with greater concerns about the consequences of COVID-19 (e.g., becoming seriously ill) because people with higher education levels may have greater engagement and interest in health information [6-8].

**Text S3. The results of accuracy and stability of estimated network using the EBIC-gLASSO approach.**

This study conducted three validations for the estimated network, including edge-weight accuracy, centrality stability, and testing for significant differences. The validation result of edge-weight accuracy was presented in S2 Fig. S2 Fig shows that Confidence Intervals (CIs) of most estimated edge-weights are small, which represents the estimated network passed the validation of edge-weight accuracy. Consequently, the interpretation for most associations between items and their strengths (i.e., edge and edge-weights) were appropriate [1]. S3 Fig shows the validation result of centrality stability. The average correlation between the maximum 75% case-dropped and original samples for both Strength and Bridge Strength I are equal or higher than 0.7, i.e., the CS-C is above 0.7 and larger than 0.5. The result shows the estimated network passed the validation of centrality stability. Consequently, the values of Strength and Bridge Strength I can remain stable after dropping large proportions (up to 75%) of the original sample.

**Text S4. The sensitivity analysis of** **anxiety and depressive symptoms regarding participants with different education levels.**

In the network comparison conducted in this study, participants were categorized into two subgroups based on their education level. The high education level subgroup included participants with a Master’s degree or above, Post-secondary education: Degree programs, or Post-secondary education: Sub-degree programs, according to a common understanding in Hong Kong. On the other hand, the low education level subgroup consists of participants with Post-secondary education: Diploma/Certificate, Secondary school education or equivalent, Primary school and below, or no formal education.

To ensure the robustness of our explanation and interpretation regarding education in the network comparison section, we performed a sensitivity analysis of anxiety and depressive symptoms using six different subgroup settings. The result of this sensitivity analysis was presented in S7 Table. Notably, a consistent finding emerged, indicating that participants with a high education level consistently exhibited higher scores on depression and anxiety, with one exception (i.e., subgroup setting_6). In this particular exception, different groups of participants demonstrated similar scores on depression and anxiety. This can be attributed to the fact that a substantial number of participants (164) belonged to the education level category of "Post-secondary education: Degree programs," which may share similar cognitive factors with participants in the "Master's degree or above" category.

By conducting this sensitivity analysis, we aimed to provide a more comprehensive understanding of the relationship between education level and anxiety and depressive symptoms in the network comparison. These findings strengthen our original conclusions and contribute to a more nuanced interpretation of the results.

**Table S1. Vulnerable subgroup setting of age, gender, education, income, marital status, employment, and residential location.**

| Demographic Feature | Subgroup | Number (percentage) | Criterion |
| --- | --- | --- | --- |
| Age | Young | 188 (50%) | Participants whose age is younger than or equal to the Median age (i.e., 34). |
|  | elder | 188 (50%) | Participants whose age is older than the Median age (i.e., 34). |
| Gender | Male | 122 (32.4%) | Participants who are male (assigned at birth). |
|  | Female | 254 (67.6%) | Participants who are female (assigned at birth). |
| Education | High level of education | 268 (71.3%) | Participants whose education level is in the range of Master’s degree or above, Post-secondary education: Degree programs, and Post-secondary education: Sub-degree programs |
|  | Low level of education | 108 (28.7%) | Participants whose education level is in the range of Post-secondary education: Diploma/Certificate, Secondary school education or equivalent, Primary school and below, and no formal education. |
| Income | High level of income | 138 (36.7%) | Participants whose income is higher than HK$ 40000. |
|  | Low level of income | 238 (63.3%) | Participants whose income is smaller than or equal to HK$ 40000. |
| Marital Status | Married | 136 (36.2%) | Participants who are married. |
|  | Unmarried | 240 (63.8%) | Participants whose marital statuses are in the range of never married, separation, divorce, and death of a spouse. |
| Employment | Employed | 253 (67.3%) | Participants whose employment statuses are in the range of the full-time job, part-time job, and self-employed. |
|  | Unemployed | 123 (32.7%) | Participants whose employment statuses are in the range of full-time student, housewife, unemployed, retired, temporary job (no contract and unstable). |
| Residential location | Hong Kong Island & Kowloon | 144 (38.3%) | Participants who live in Hong Kong Island and Kowloon. |
|  | New Territories | 232 (61.7%) | Participants who live in New Territories. |

**Table S2. The results of reliability and validity tests of questionnaires for people’s daily activities, people’s perceptions of COVID-19, and people’s perceptions of neighborhood cohesion.**

S2.1. The results of Cronbach's alpha, KMO, and Bartlett's Tests.

| Category | Cronbach's alpha | KMO | Sig. of Bartlett's Test |
| --- | --- | --- | --- |
| Perceptions of COVID-19 | 0.703 | 0.642 | <.001 |
| Perceptions of neighborhood cohesion | 0.905 | 0.877 | 0 |
| Daily activities | 0.642 | 0.766 | <.001 |
| Perceptions of COVID-19, Perceptions of neighborhood cohesion, and Daily activities | 0.737 | 0.801 | 0 |

Notes:

1. According to the general rule of thumb for Cronbach's alpha, a value of 0.70 or higher is typically considered indicative of good internal consistency. In the context of our study, the questionnaires assessing perceptions of COVID-19 and perceptions of neighborhood cohesion demonstrated good reliability, as their Cronbach's alpha values exceeded 0.70. However, it is worth noting that the questionnaire pertaining to daily activities exhibited a slightly lower reliability, with a Cronbach's alpha of 0.642. While this value is below the recommended threshold of 0.70, it is still considered acceptable or tolerant in some cases.

2. Factor analysis was conducted to assess the construct validity of the questionnaires in this study. The KMO and Bartlett's tests were used as criteria for further analysis. The questionnaires assessing perceptions of COVID-19, perceptions of neighborhood cohesion, daily activities, and the combined questionnaire passed both tests, indicating their suitability for factor analysis. Although the KMO value for the perceptions of COVID-19 questionnaire was slightly below the recommended threshold (0.7), , it is still considered acceptable or tolerant in some cases.

S2.2. Results of factor analysis of the perceptions of COVID-19 questionnaire, including the rotated component matrix and total variance explained.

|  | Component |  |
| --- | --- | --- |
|  | 1 | 2 |
| PC1 | -0.1 | 0.775 |
| PC2 | 0.214 | 0.796 |
| PC3 | 0.136 | 0.623 |
| PC4 | 0.584 | 0.479 |
| PC5 | 0.88 |  |
| PC6 | 0.907 |  |
| Total variance explained | | 64.509% |

S2.3. Results of factor analysis of the perceptions of neighborhood cohesion questionnaire, including the rotated component matrix and total variance explained.

|  | Component |  |
| --- | --- | --- |
|  | 1 | 2 |
| PN1 | 0.84 | 0.196 |
| PN2 | 0.824 | 0.189 |
| PN3 | 0.849 | 0.255 |
| PN4 | 0.799 | 0.343 |
| PN5 | 0.818 | 0.332 |
| PN6 | 0.239 | 0.856 |
| PN7 | 0.308 | 0.786 |
| PN8 | 0.223 | 0.879 |
| Total variance explained | | 76.327% |

S2.4. Results of factor analysis of the daily activities questionnaire, including the rotated component matrix and total variance explained.

|  | Component |  |  |
| --- | --- | --- | --- |
|  | 1 | 2 | 3 |
| DA1 | 0.489 | 0.357 | 0.104 |
| DA2 |  | 0.636 | -0.166 |
| DA3 | 0.236 | 0.27 | -0.601 |
| DA4 | 0.109 | 0.73 |  |
| DA5 | 0.87 |  |  |
| DA6 | 0.894 | 0.141 |  |
| DA7 | 0.832 |  | -0.137 |
| DA8 |  |  | 0.748 |
| DA9 | 0.219 | 0.49 | 0.309 |
| Total variance explained | | | 56.614% |

S2.5. Results of factor analysis of the combination of perceptions of COVID-19, perceptions of neighborhood cohesion, and daily activities questionnaires, including the rotated component matrix and total variance explained.

|  | Component |  |  |  |  |  |  |
| --- | --- | --- | --- | --- | --- | --- | --- |
|  | 1 | 2 | 3 | 4 | 5 | 6 | 7 |
| PC1 |  | 0.193 |  |  | 0.763 | -0.115 | 0.173 |
| PC2 |  |  |  | 0.291 | 0.725 | 0.141 |  |
| PC3 |  |  |  | 0.123 | 0.39 | 0.614 |  |
| PC4 |  |  |  | 0.534 | 0.418 | 0.415 | -0.115 |
| PC5 |  |  |  | 0.853 |  |  |  |
| PC6 |  |  |  | 0.871 |  |  |  |
| PN1 | 0.855 |  | 0.123 |  |  |  |  |
| PN2 | 0.841 |  | 0.113 |  |  |  |  |
| PN3 | 0.861 |  | 0.201 |  |  |  |  |
| PN4 | 0.814 |  | 0.286 |  |  |  |  |
| PN5 | 0.833 |  | 0.281 |  |  |  |  |
| PN6 | 0.304 |  | 0.833 |  |  |  |  |
| PN7 | 0.372 |  | 0.754 |  |  | -0.106 |  |
| PN8 | 0.291 |  | 0.851 |  |  |  |  |
| DA1 | 0.194 | 0.394 |  | -0.238 | 0.424 | -0.152 | 0.183 |
| DA2 |  |  |  |  |  |  | 0.739 |
| DA3 |  | 0.232 | 0.118 | 0.12 |  |  | 0.586 |
| DA4 |  |  |  |  | 0.456 |  | 0.496 |
| DA5 |  | 0.86 |  | -0.125 |  |  |  |
| DA6 |  | 0.888 |  |  | 0.16 |  |  |
| DA7 |  | 0.847 |  |  |  |  | 0.102 |
| DA8 |  |  |  |  | 0.248 | -0.741 | -0.153 |
| DA9 |  | 0.194 |  | -0.387 | 0.319 | 0.346 | -0.101 |
| Total variance explained | | | | | | | 67.697 |

Notes:

1. The extraction method employed in S7.2-7.5 is principal component analysis, while the rotation method is varimax with Kaiser normalization.

2. The rotated component matrixes and total variances explained in S7.2-7.5 provided evidence of construct validity for the questionnaires of perceptions of COVID-19, perceptions of neighborhood cohesion, and daily activities.

**Table S3. The weighted adjacency matrix storing the values of associations for each edge.**

|  | AN.1 | AN.2 | DE.1 | DE.2 | PC.1 | PC.2 | PC.3 | PC.4 | PC.5 | PC.6 | PN.1 | PN.2 | PN.3 | PN.4 | PN.5 | PN.6 | PN.7 | PN.8 | DA.1 | DA.2 | DA.3 | DA.4 | DA.5 | DA.6 | DA.7 | DA.8 | DA.9 |
| --- | --- | --- | --- | --- | --- | --- | --- | --- | --- | --- | --- | --- | --- | --- | --- | --- | --- | --- | --- | --- | --- | --- | --- | --- | --- | --- | --- |
| AN.1 | 0.000 | 0.411 | 0.195 | 0.076 | 0.099 | 0.174 | 0.007 | 0.013 | 0.000 | 0.000 | 0.000 | 0.000 | 0.000 | 0.004 | 0.000 | 0.000 | 0.000 | 0.000 | 0.000 | 0.000 | 0.000 | 0.023 | 0.000 | 0.000 | 0.035 | 0.000 | 0.000 |
| AN.2 | 0.411 | 0.000 | 0.320 | 0.157 | 0.000 | 0.090 | 0.000 | 0.000 | 0.000 | 0.000 | 0.000 | 0.000 | 0.000 | 0.000 | 0.000 | 0.000 | 0.000 | 0.000 | 0.000 | 0.000 | 0.000 | 0.000 | 0.000 | 0.000 | 0.000 | 0.000 | 0.000 |
| DE.1 | 0.195 | 0.320 | 0.000 | 0.357 | 0.052 | 0.002 | 0.000 | 0.000 | 0.026 | 0.000 | 0.000 | 0.000 | 0.000 | 0.000 | 0.000 | 0.000 | 0.000 | 0.000 | 0.000 | 0.000 | 0.000 | 0.000 | 0.000 | 0.000 | 0.000 | 0.028 | 0.000 |
| DE.2 | 0.076 | 0.157 | 0.357 | 0.000 | 0.122 | 0.000 | 0.000 | 0.000 | 0.000 | 0.000 | 0.006 | 0.022 | 0.000 | 0.023 | 0.000 | 0.000 | 0.009 | 0.000 | 0.000 | 0.000 | 0.067 | 0.072 | 0.000 | 0.000 | 0.000 | 0.006 | 0.000 |
| PC.1 | 0.099 | 0.000 | 0.052 | 0.122 | 0.000 | 0.236 | 0.027 | 0.000 | 0.000 | 0.000 | 0.000 | 0.000 | 0.000 | 0.000 | 0.000 | 0.000 | 0.000 | 0.000 | 0.245 | 0.000 | 0.026 | 0.089 | 0.011 | 0.033 | 0.000 | 0.000 | 0.027 |
| PC.2 | 0.174 | 0.090 | 0.002 | 0.000 | 0.236 | 0.000 | 0.075 | 0.261 | 0.019 | 0.000 | 0.000 | 0.000 | 0.000 | 0.000 | 0.000 | 0.000 | 0.000 | 0.000 | 0.000 | 0.000 | 0.000 | 0.016 | 0.000 | 0.001 | 0.014 | 0.000 | 0.000 |
| PC.3 | 0.007 | 0.000 | 0.000 | 0.000 | 0.027 | 0.075 | 0.000 | 0.197 | 0.000 | 0.000 | 0.000 | 0.000 | 0.000 | 0.000 | 0.000 | 0.000 | 0.000 | 0.000 | 0.000 | 0.025 | 0.000 | 0.015 | 0.008 | 0.027 | 0.000 | -0.123 | 0.046 |
| PC.4 | 0.013 | 0.000 | 0.000 | 0.000 | 0.000 | 0.261 | 0.197 | 0.000 | 0.112 | 0.156 | 0.000 | -0.021 | 0.000 | 0.000 | 0.000 | 0.000 | -0.016 | 0.000 | 0.000 | 0.000 | 0.000 | 0.000 | 0.000 | 0.000 | 0.000 | 0.000 | 0.000 |
| PC.5 | 0.000 | 0.000 | 0.026 | 0.000 | 0.000 | 0.019 | 0.000 | 0.112 | 0.000 | 0.602 | 0.000 | 0.000 | 0.000 | 0.000 | 0.000 | 0.000 | 0.000 | -0.018 | -0.044 | 0.000 | 0.000 | 0.000 | 0.000 | 0.000 | 0.000 | 0.000 | 0.000 |
| PC.6 | 0.000 | 0.000 | 0.000 | 0.000 | 0.000 | 0.000 | 0.000 | 0.156 | 0.602 | 0.000 | 0.000 | 0.000 | 0.000 | 0.000 | 0.000 | 0.000 | 0.000 | -0.005 | -0.013 | 0.000 | 0.000 | 0.000 | -0.033 | 0.000 | 0.000 | 0.000 | -0.037 |
| PN.1 | 0.000 | 0.000 | 0.000 | 0.006 | 0.000 | 0.000 | 0.000 | 0.000 | 0.000 | 0.000 | 0.000 | 0.357 | 0.180 | 0.200 | 0.096 | 0.000 | 0.005 | 0.000 | 0.000 | 0.000 | 0.000 | 0.000 | 0.000 | 0.000 | 0.000 | 0.000 | 0.000 |
| PN.2 | 0.000 | 0.000 | 0.000 | 0.022 | 0.000 | 0.000 | 0.000 | -0.021 | 0.000 | 0.000 | 0.357 | 0.000 | 0.188 | 0.000 | 0.164 | 0.000 | 0.031 | 0.043 | 0.056 | 0.000 | 0.036 | 0.000 | 0.000 | 0.000 | 0.000 | 0.000 | 0.000 |
| PN.3 | 0.000 | 0.000 | 0.000 | 0.000 | 0.000 | 0.000 | 0.000 | 0.000 | 0.000 | 0.000 | 0.180 | 0.188 | 0.000 | 0.258 | 0.251 | 0.058 | 0.000 | 0.000 | 0.000 | 0.000 | 0.000 | 0.000 | 0.000 | 0.000 | 0.000 | 0.000 | 0.000 |
| PN.4 | 0.004 | 0.000 | 0.000 | 0.023 | 0.000 | 0.000 | 0.000 | 0.000 | 0.000 | 0.000 | 0.200 | 0.000 | 0.258 | 0.000 | 0.309 | 0.049 | 0.181 | 0.000 | 0.000 | 0.000 | 0.000 | 0.000 | 0.000 | 0.000 | 0.000 | 0.000 | 0.000 |
| PN.5 | 0.000 | 0.000 | 0.000 | 0.000 | 0.000 | 0.000 | 0.000 | 0.000 | 0.000 | 0.000 | 0.096 | 0.164 | 0.251 | 0.309 | 0.000 | 0.077 | 0.000 | 0.085 | 0.008 | 0.000 | 0.000 | 0.000 | 0.000 | 0.000 | 0.000 | 0.000 | -0.005 |
| PN.6 | 0.000 | 0.000 | 0.000 | 0.000 | 0.000 | 0.000 | 0.000 | 0.000 | 0.000 | 0.000 | 0.000 | 0.000 | 0.058 | 0.049 | 0.077 | 0.000 | 0.161 | 0.458 | 0.000 | 0.000 | 0.000 | 0.000 | 0.000 | 0.000 | 0.000 | 0.000 | 0.000 |
| PN.7 | 0.000 | 0.000 | 0.000 | 0.009 | 0.000 | 0.000 | 0.000 | -0.016 | 0.000 | 0.000 | 0.005 | 0.031 | 0.000 | 0.181 | 0.000 | 0.161 | 0.000 | 0.280 | 0.001 | 0.000 | 0.023 | 0.000 | 0.000 | 0.000 | 0.000 | 0.000 | 0.000 |
| PN.8 | 0.000 | 0.000 | 0.000 | 0.000 | 0.000 | 0.000 | 0.000 | 0.000 | -0.018 | -0.005 | 0.000 | 0.043 | 0.000 | 0.000 | 0.085 | 0.458 | 0.280 | 0.000 | 0.024 | 0.000 | 0.000 | 0.000 | 0.000 | 0.000 | 0.000 | 0.000 | 0.000 |
| DA.1 | 0.000 | 0.000 | 0.000 | 0.000 | 0.245 | 0.000 | 0.000 | 0.000 | -0.044 | -0.013 | 0.000 | 0.056 | 0.000 | 0.000 | 0.008 | 0.000 | 0.001 | 0.024 | 0.000 | 0.036 | 0.000 | 0.002 | 0.244 | 0.040 | 0.000 | 0.000 | 0.000 |
| DA.2 | 0.000 | 0.000 | 0.000 | 0.000 | 0.000 | 0.000 | 0.025 | 0.000 | 0.000 | 0.000 | 0.000 | 0.000 | 0.000 | 0.000 | 0.000 | 0.000 | 0.000 | 0.000 | 0.036 | 0.000 | 0.014 | 0.044 | 0.020 | 0.000 | 0.000 | 0.000 | 0.030 |
| DA.3 | 0.000 | 0.000 | 0.000 | 0.067 | 0.026 | 0.000 | 0.000 | 0.000 | 0.000 | 0.000 | 0.000 | 0.036 | 0.000 | 0.000 | 0.000 | 0.000 | 0.023 | 0.000 | 0.000 | 0.014 | 0.000 | 0.104 | 0.030 | 0.000 | 0.097 | 0.000 | 0.000 |
| DA.4 | 0.023 | 0.000 | 0.000 | 0.072 | 0.089 | 0.016 | 0.015 | 0.000 | 0.000 | 0.000 | 0.000 | 0.000 | 0.000 | 0.000 | 0.000 | 0.000 | 0.000 | 0.000 | 0.002 | 0.044 | 0.104 | 0.000 | 0.000 | 0.063 | 0.000 | 0.000 | 0.100 |
| DA.5 | 0.000 | 0.000 | 0.000 | 0.000 | 0.011 | 0.000 | 0.008 | 0.000 | 0.000 | -0.033 | 0.000 | 0.000 | 0.000 | 0.000 | 0.000 | 0.000 | 0.000 | 0.000 | 0.244 | 0.020 | 0.030 | 0.000 | 0.000 | 0.452 | 0.147 | 0.000 | 0.074 |
| DA.6 | 0.000 | 0.000 | 0.000 | 0.000 | 0.033 | 0.001 | 0.027 | 0.000 | 0.000 | 0.000 | 0.000 | 0.000 | 0.000 | 0.000 | 0.000 | 0.000 | 0.000 | 0.000 | 0.040 | 0.000 | 0.000 | 0.063 | 0.452 | 0.000 | 0.398 | 0.000 | 0.226 |
| DA.7 | 0.035 | 0.000 | 0.000 | 0.000 | 0.000 | 0.014 | 0.000 | 0.000 | 0.000 | 0.000 | 0.000 | 0.000 | 0.000 | 0.000 | 0.000 | 0.000 | 0.000 | 0.000 | 0.000 | 0.000 | 0.097 | 0.000 | 0.147 | 0.398 | 0.000 | 0.000 | 0.030 |
| DA.8 | 0.000 | 0.000 | 0.028 | 0.006 | 0.000 | 0.000 | -0.123 | 0.000 | 0.000 | 0.000 | 0.000 | 0.000 | 0.000 | 0.000 | 0.000 | 0.000 | 0.000 | 0.000 | 0.000 | 0.000 | 0.000 | 0.000 | 0.000 | 0.000 | 0.000 | 0.000 | 0.000 |
| DA.9 | 0.000 | 0.000 | 0.000 | 0.000 | 0.027 | 0.000 | 0.046 | 0.000 | 0.000 | -0.037 | 0.000 | 0.000 | 0.000 | 0.000 | -0.005 | 0.000 | 0.000 | 0.000 | 0.000 | 0.030 | 0.000 | 0.100 | 0.074 | 0.226 | 0.030 | 0.000 | 0.000 |

**Table S4. Original values of Strength, Bridge Strength I, and Bridge Strength II, and the descriptive statistics of each item in the estimated network.**

| Items | Mean | SD | Strength | Bridge Strength I | Bridge Strength II | Skewness | Kurtosis |
| --- | --- | --- | --- | --- | --- | --- | --- |
| AN.1 | 2.753 | 1.150 | 1.036 | 0.625 | 0.681 | -0.474 | 0.313 |
| AN.2 | 2.306 | 1.286 | 0.978 | 0.567 | 0.888 | -0.026 | -0.414 |
| DA.1 | 4.649 | 1.442 | 0.712 | 0.391 | 0.000 | -0.934 | 0.058 |
| DA.2 | 0.505 | 1.164 | 0.169 | 0.025 | 0.000 | 0.053 | 1.803 |
| DA.3 | 0.468 | 1.287 | 0.397 | 0.152 | 0.067 | 0.048 | 0.090 |
| DA.4 | 1.205 | 1.168 | 0.529 | 0.215 | 0.096 | -0.391 | 0.299 |
| DA.5 | 2.186 | 1.238 | 1.019 | 0.052 | 0.000 | -2.061 | 4.620 |
| DA.6 | 2.210 | 1.118 | 1.241 | 0.061 | 0.000 | -1.813 | 3.682 |
| DA.7 | 1.984 | 1.215 | 0.721 | 0.049 | 0.035 | -1.294 | 1.366 |
| DA.8 | 1.282 | 1.459 | 0.157 | 0.157 | 0.034 | 1.237 | 1.145 |
| DA.9 | 1.824 | 1.658 | 0.575 | 0.114 | 0.000 | -1.689 | 2.012 |
| DE.1 | 2.330 | 1.350 | 0.980 | 0.622 | 0.873 | -0.073 | -0.642 |
| DE.2 | 2.758 | 1.251 | 0.917 | 0.559 | 0.590 | -0.206 | -0.275 |
| PC.1 | 4.303 | 1.114 | 0.967 | 0.704 | 0.273 | -0.823 | 1.128 |
| PC.2 | 1.957 | 1.123 | 0.886 | 0.296 | 0.265 | 0.331 | -0.805 |
| PC.3 | 4.346 | 1.467 | 0.549 | 0.251 | 0.007 | -0.538 | -0.299 |
| PC.4 | 3.324 | 1.405 | 0.777 | 0.050 | 0.013 | -0.097 | -0.039 |
| PC.5 | 1.612 | 1.291 | 0.820 | 0.088 | 0.026 | 0.880 | 0.870 |
| PC.6 | 1.404 | 1.171 | 0.845 | 0.087 | 0.000 | 0.955 | 1.345 |
| PN.1 | 2.330 | 0.984 | 0.843 | 0.006 | 0.006 | 0.646 | 0.885 |
| PN.2 | 2.606 | 1.030 | 0.919 | 0.136 | 0.022 | 0.459 | -0.073 |
| PN.3 | 2.410 | 0.948 | 0.935 | 0.000 | 0.000 | 0.794 | 0.775 |
| PN.4 | 2.306 | 0.918 | 1.024 | 0.027 | 0.027 | 0.889 | 1.403 |
| PN.5 | 2.527 | 0.968 | 0.995 | 0.012 | 0.000 | 0.531 | 0.409 |
| PN.6 | 2.737 | 1.047 | 0.803 | 0.000 | 0.000 | 0.316 | 0.083 |
| PN.7 | 2.340 | 0.939 | 0.707 | 0.048 | 0.009 | 0.649 | 1.263 |
| PN.8 | 2.814 | 1.077 | 0.914 | 0.047 | 0.000 | 0.182 | 0.133 |

**Table S5. The results of edge invariance test based on education. Here, presented edges are different at a significant level of 0.053 (i.e., p-value = 0.053).**

| **The nodes that the edge lies between** | | **P-values for people with high level of education and low level of education** |
| --- | --- | --- |
| PC.5 | PC.6 | 0.002 |
| AN.1 | PC.6 | 0.010 |
| PC.2 | PC.4 | 0.018 |
| DA.1 | DA.5 | 0.024 |
| PN.1 | PN.5 | 0.030 |
| DA.7 | DA.9 | 0.033 |
| DE.1 | DA.3 | 0.034 |
| PC.1 | DA.1 | 0.050 |
| AN.1 | DA.4 | 0.050 |
| DE.2 | PN.4 | 0.053 |

**Table S6. The statistics of self-reported anxiety and depressive symptoms between the high-education group and the low-education group.**

| Statistical Indicator | High-education subgroup | | | | Low-education subgroup | | | |
| --- | --- | --- | --- | --- | --- | --- | --- | --- |
|  | AN.1 | AN.2 | DE.1 | DE.2 | AN.1 | AN.2 | DE.1 | DE.2 |
| count | 268 | 268 | 268 | 268 | 108 | 108 | 108 | 108 |
| mean | 2.81 | 2.37 | 2.39 | 2.87 | 2.61 | 2.15 | 2.19 | 2.48 |
| std | 1.10 | 1.26 | 1.35 | 1.24 | 1.27 | 1.34 | 1.35 | 1.24 |
| min | 0 | 0 | 0 | 0 | 0 | 0 | 0 | 0 |
| 25% | 2 | 2 | 1 | 2 | 2 | 1 | 1 | 2 |
| 50% | 3 | 2 | 3 | 3 | 3 | 2 | 2 | 3 |
| 75% | 3 | 3 | 3 | 4 | 3 | 3 | 3 | 3 |
| max | 5 | 5 | 5 | 5 | 5 | 5 | 5 | 5 |

**Table S7. The mean scores of anxiety and depressive symptoms between the high-education group and the low-education group under six different subgroup settings.**

|  | High education level | | | | Low education level | | | |
| --- | --- | --- | --- | --- | --- | --- | --- | --- |
|  | AN.1 | AN.2 | DE.1 | DE.2 | AN.1 | AN.2 | DE.1 | DE.2 |
| Subgroup setting_1^a^ | 2.76 | 2.31 | 2.34 | 2.76 | 1.50 | 1.50 | 0.00 | 2.50 |
| Subgroup setting_2^b^ | 2.76 | 2.31 | 2.34 | 2.76 | 2.25 | 2.25 | 1.75 | 3.00 |
| Subgroup setting_3^c^ | 2.81 | 2.35 | 2.38 | 2.85 | 2.56 | 2.15 | 2.14 | 2.41 |
| Subgroup setting_4^d^ | 2.81 | 2.37 | 2.39 | 2.87 | 2.61 | 2.15 | 2.19 | 2.48 |
| Subgroup setting_5^e^ | 2.80 | 2.37 | 2.40 | 2.87 | 2.66 | 2.16 | 2.18 | 2.51 |
| Subgroup setting_6^f^ | 2.69 | 2.14 | 2.06 | 2.77 | 2.77 | 2.36 | 2.42 | 2.75 |

Notes:

a. In subgroup setting_1, participants were divided into high- and low-education-level groups. The low- education-level group consisted of participants with “no formal education”, while the high-education-level group included participants with “master’s degree or above”, “post-secondary education: degree programs”, “post-secondary education: sub-degree programs”, “post-secondary education: diploma/certificate”, “secondary school education or equivalent”, or “primary school and below”.

b. In subgroup setting_2, participants were divided into high- and low-education-level groups. The low- education-level group consisted of participants with “no formal education” or “primary school and below”, while the high-education-level group included participants with “master’s degree or above”, “post-secondary education: degree programs”, “post-secondary education: sub-degree programs”, “post-secondary education: diploma/certificate”, or “secondary school education or equivalent”.

c. In subgroup setting_3, participants were divided into high- and low-education-level groups. The low- education-level- group consisted of participants with “no formal education”, “primary school and below”, or “secondary school education or equivalent”, while the high-education-level group included participants with “master’s degree or above”, “post-secondary education: degree programs”, “post-secondary education: sub-degree programs”, or “post-secondary education: diploma/certificate”.

d. In subgroup setting_4, participants were divided into high- and low-education-level groups. The low- education-level group consisted of participants with “no formal education”, “primary school and below”, “secondary school education or equivalent”, or “post-secondary education: diploma/certificate”, while the high-education-level group included participants with “master’s degree or above”, “post-secondary education: degree programs”, or “post-secondary education: sub-degree programs”.

e. In subgroup setting_5, participants were divided into high- and low-education-level groups. The low-education-level group consisted of participants with “no formal education”, “primary school and below”, “secondary school education or equivalent”, “post-secondary education: diploma/certificate”, or “post-secondary education: sub-degree programs”, while the high-education-level group included participants with “master’s degree or above” or “post-secondary education: degree programs”.

f. In subgroup setting_6, participants were divided into high- and low-education-level groups. The low- education-level group consisted of participants with “no formal education”, “primary school and below”, “secondary school education or equivalent”, “post-secondary education: diploma/certificate”, “post-secondary education: sub-degree programs”, or “post-secondary education: degree programs”, while the high-education- level group included participants with “master’s degree or above”.

**Fig S1. The distributions of four anxiety and depression items for different subgroups based on** **age (a), gender (b), education (c), income (d), marital status (e), employment (f), and residential location (g). The subfig (h) describes the distributions of four anxiety and depression items for the whole sample.** Four items on the horizontal axis represent different measuring questions of anxiety and depression: AN-1: Feeling nervous, anxious, or on edge; AN-2: Not being able to stop or control worrying; DE-1: Feeling down, depressed, or hopeless; DE-2: Little interest or pleasure in doing things. The numbers on the vertical axis represent the extent of frequency that individual perceived anxiety or depression symptoms in 2 weeks: 0 is never; 1 is almost none, 2 is very few, 3 is sometimes, 4 is often, 5 is always. The height of the boundary represents the probability of each frequency.


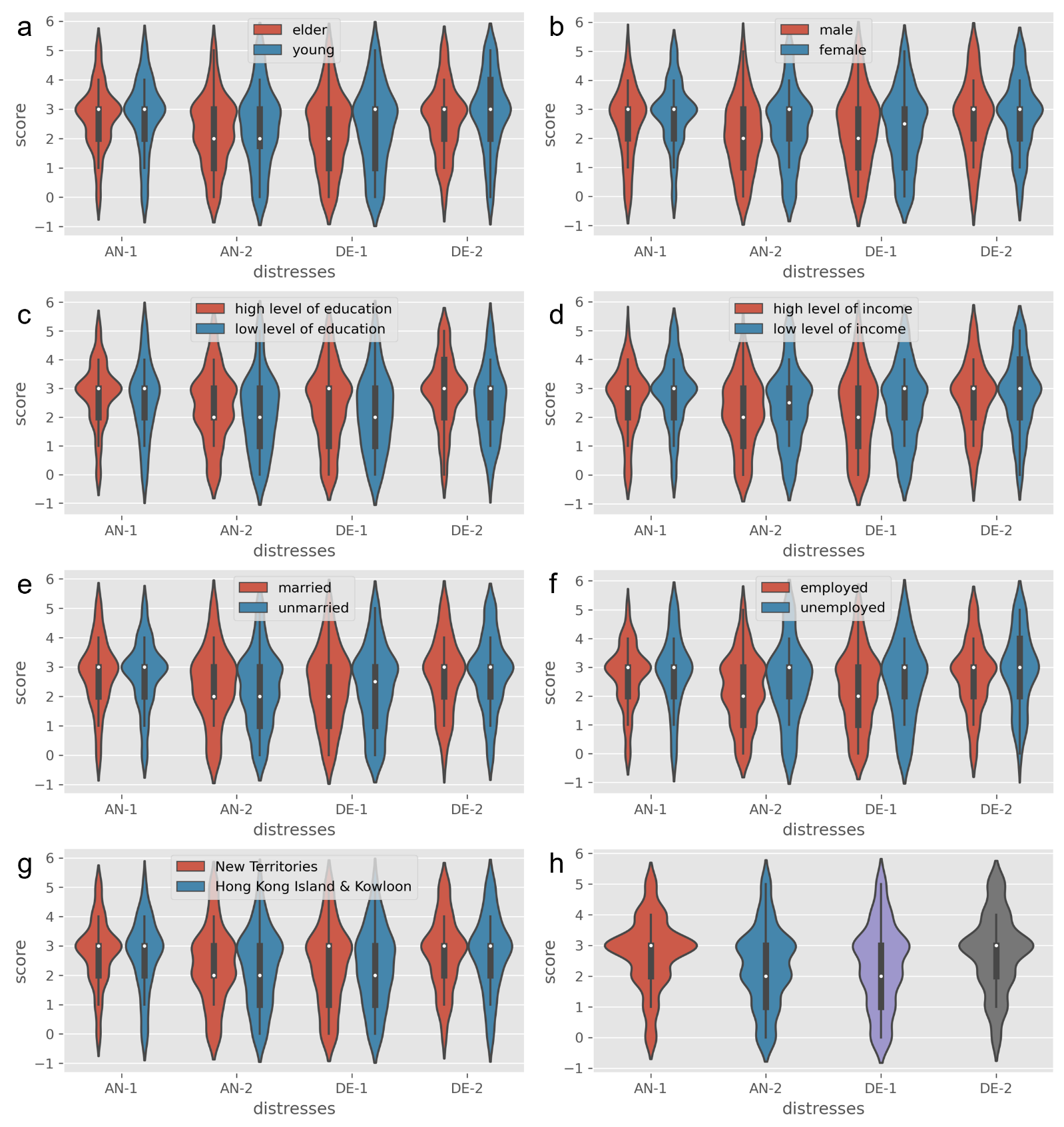


**Fig S2. Bootstrapped confidence intervals (CIs) of estimated edge-weights for the estimated network.**

**
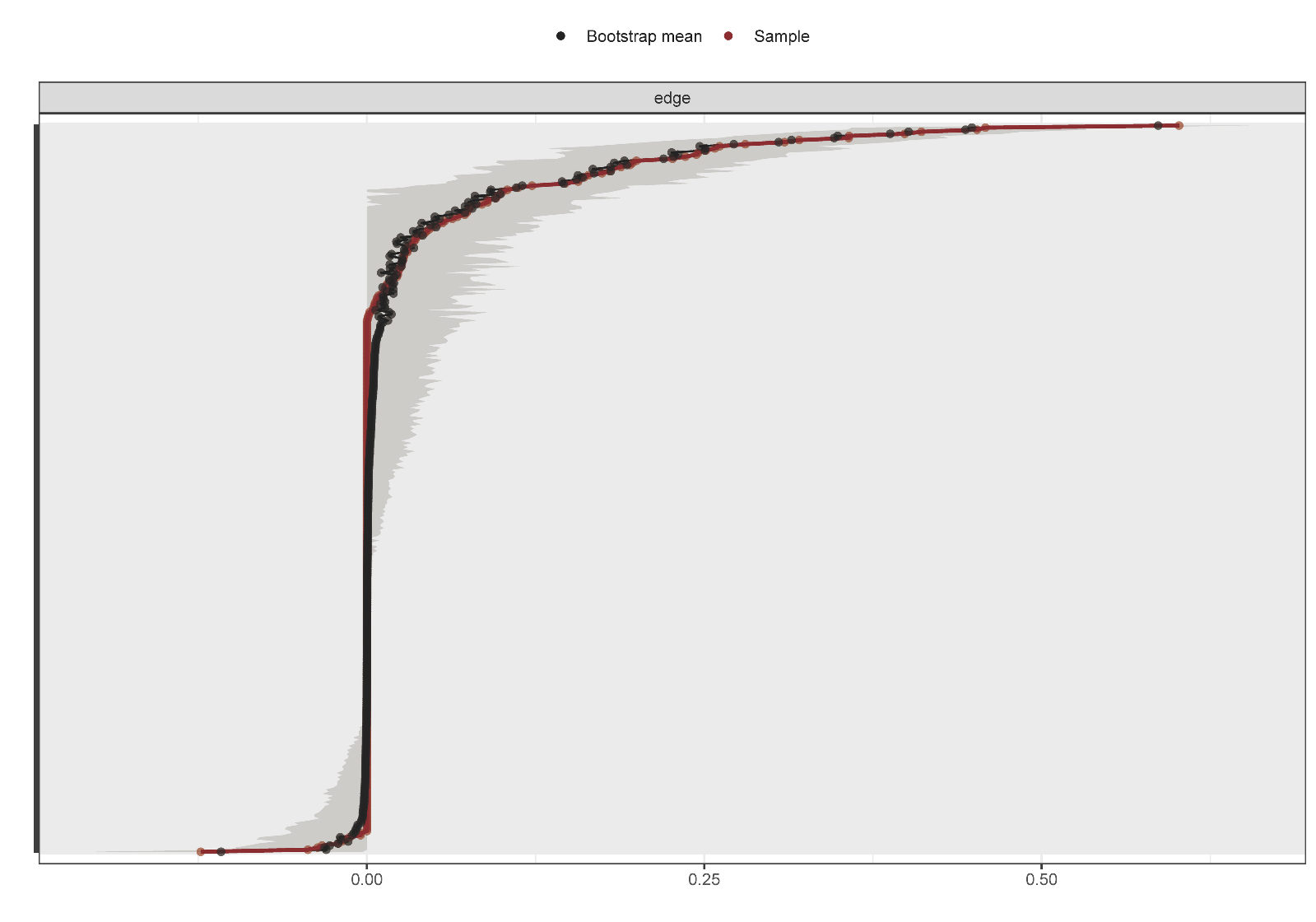
**

**Fig S3. Average correlations between strength and Bridge Strength I of networks sampled with case-dropped samples and the original sample.**

**
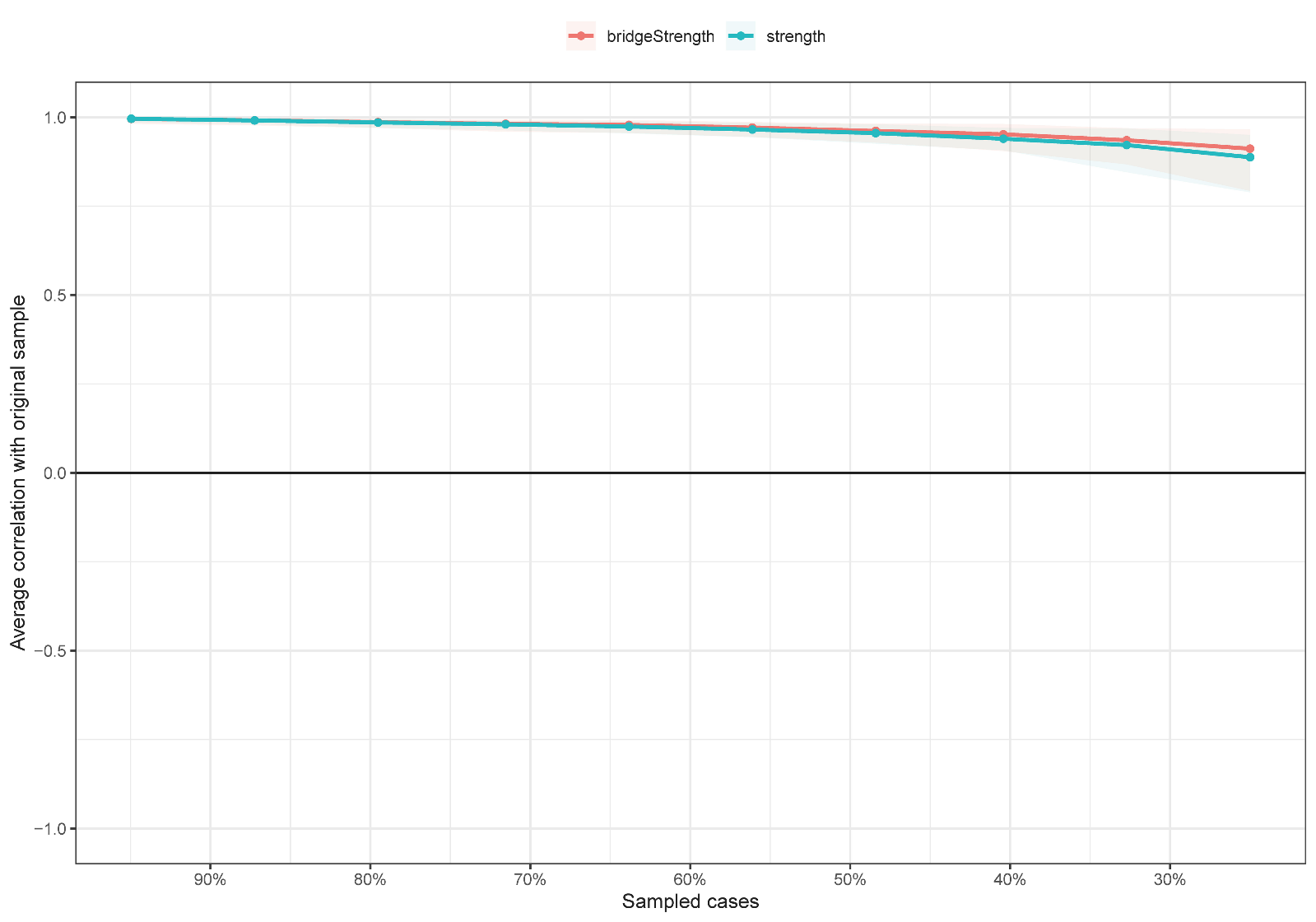
**

**Fig S4.** **The results of network invariance tests and global strength invariance tests based on (a) age, (b) gender, (c) education, (d) income, (e) marital status, (f) employment, and (g) residential location.** The grey bars indicate the reference distribution under the null hypothesis generated from the permutation procedure. The red triangle indicates the observed maximum difference in paired edge weights (left panel) and the observed difference in global strength between networks of subgroups (right panel). P values on the top indicate whether the network and global strength invariance tests are significant. Here, thresholds of p-value are 0.058 and 0.045 for network invariance test and global strength invariance test, respectively.


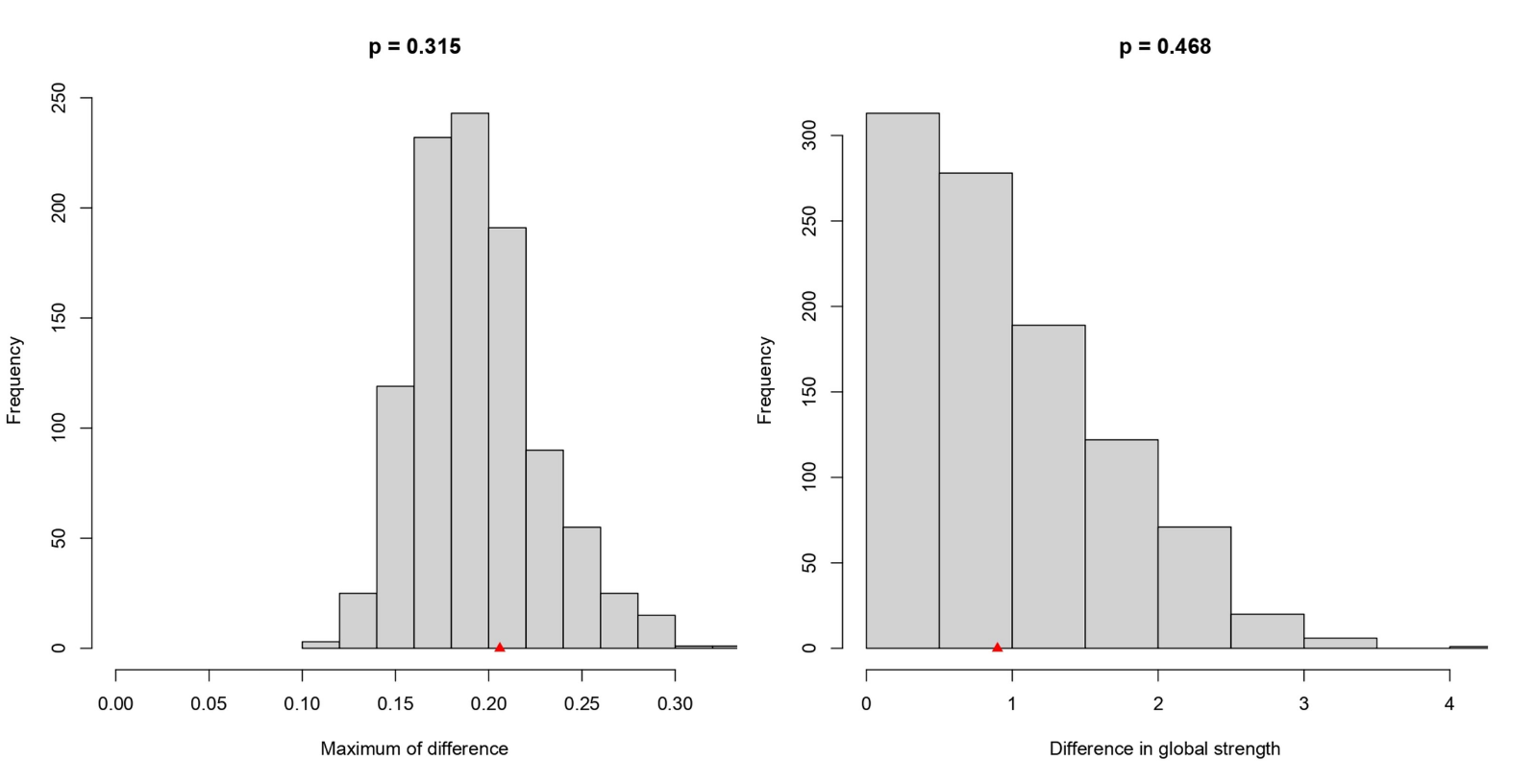


(a) Network invariance test and global strength test between young people (N=188) and elder people (N=188).


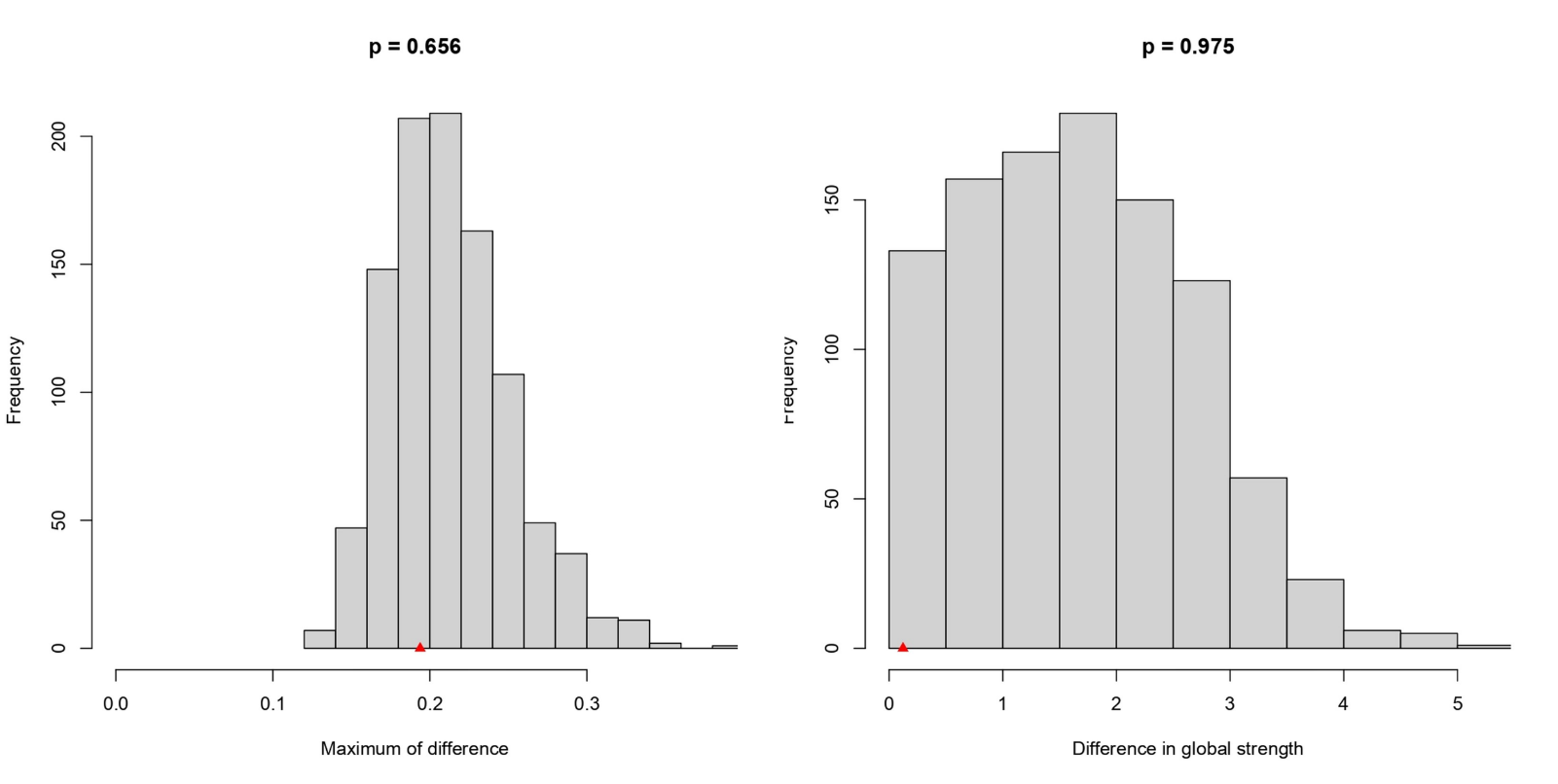


(b) Network invariance test and global strength test between males (N=122) and females (N=254).


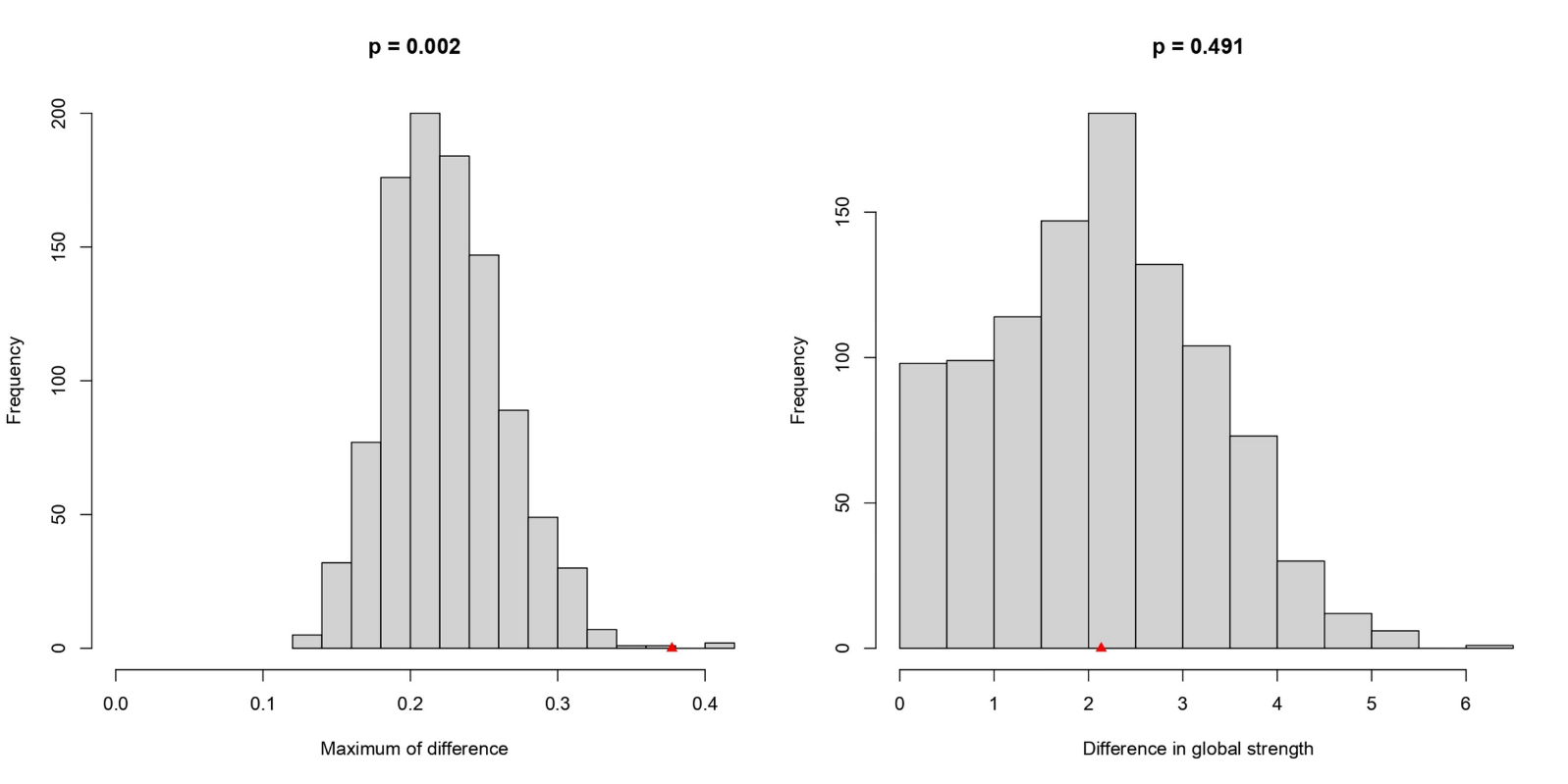


(c) Network invariance test and global strength test between people with low level of education (N=108) and people with high level of education (N=268).


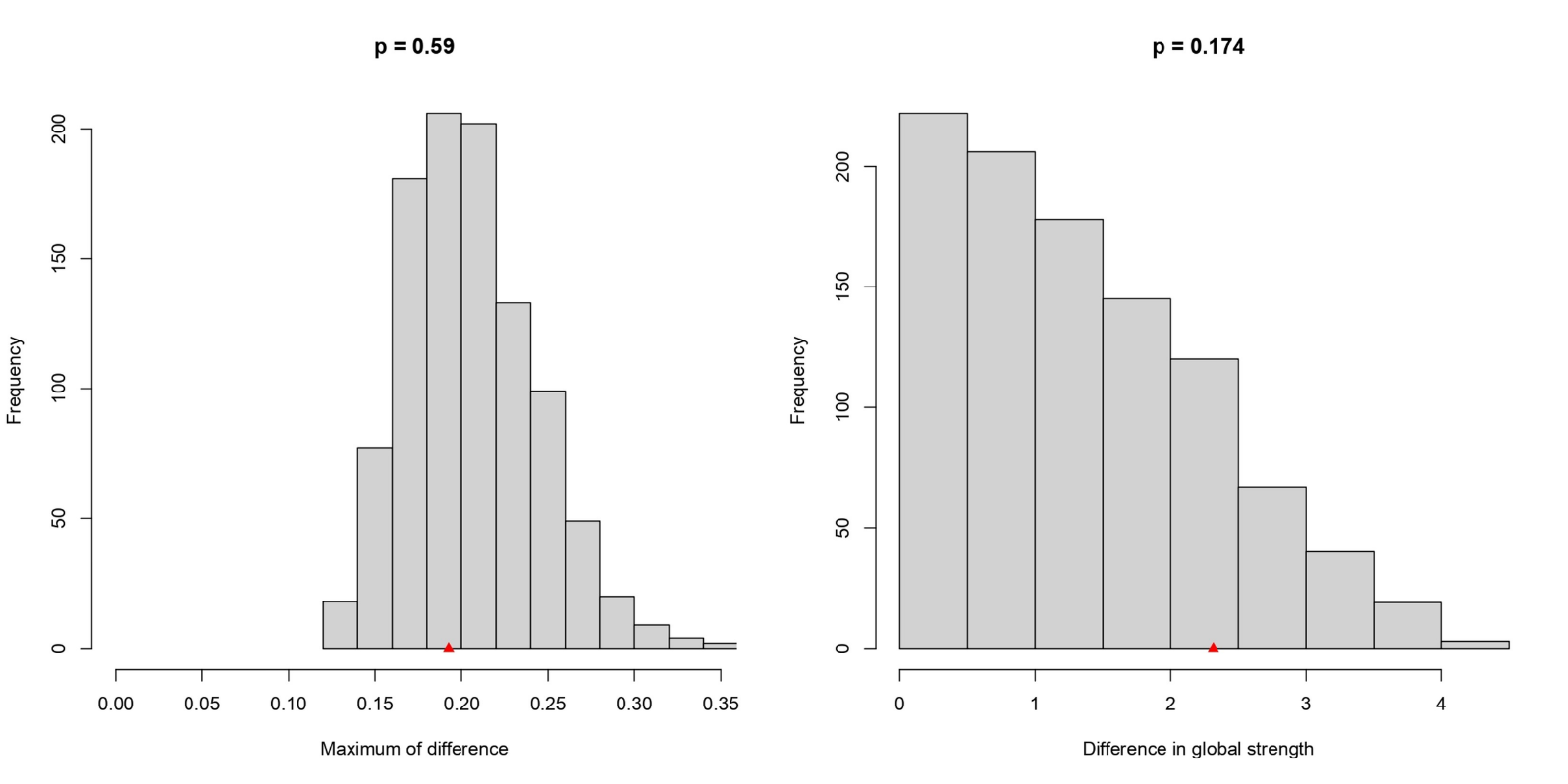


(d) Network invariance test and global strength test between people with low level of income (N=138) and people with high level of income (N=238).


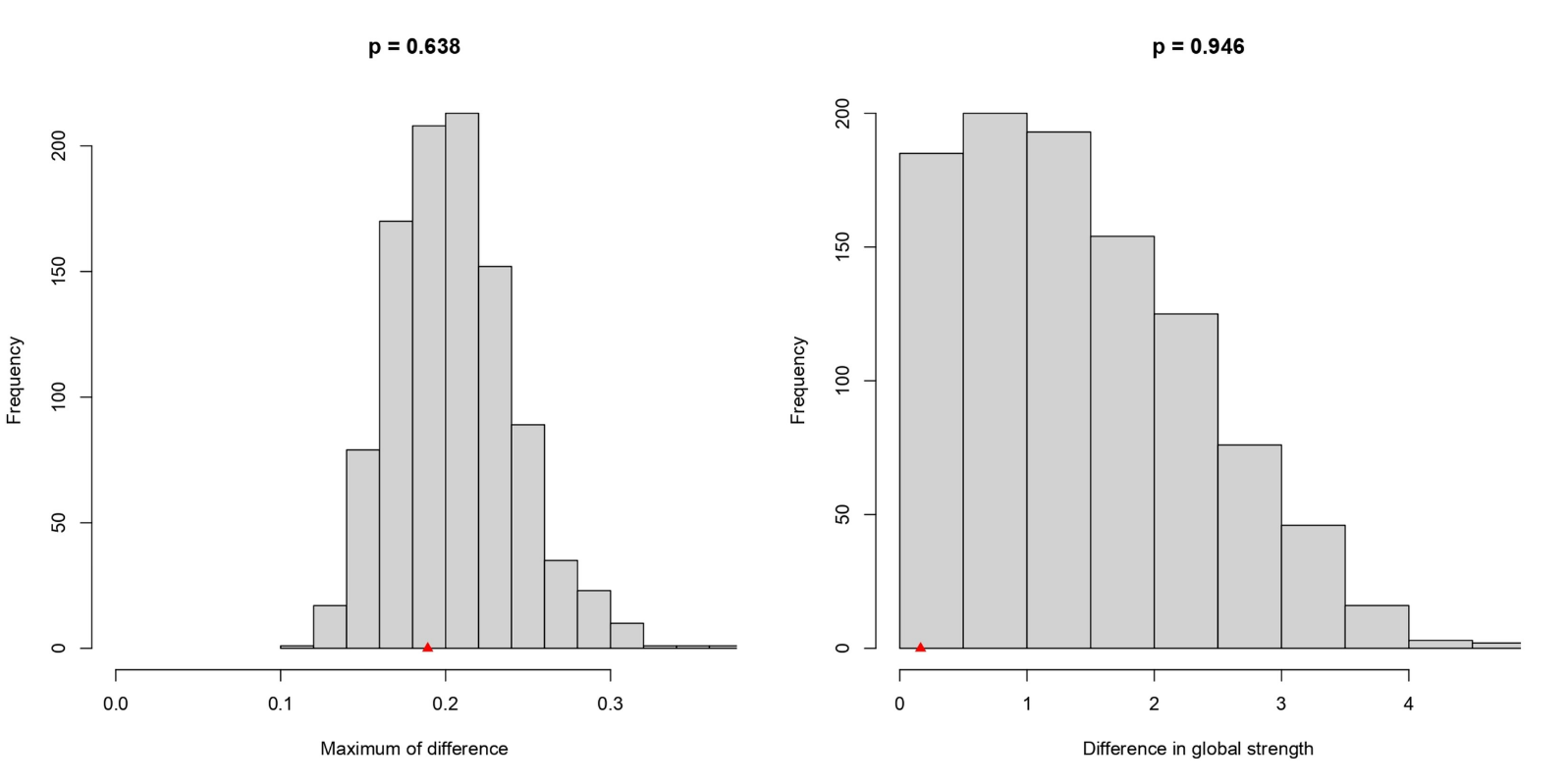


(e) Network invariance test and global strength test between married (N=136) and unmarried (N=240).


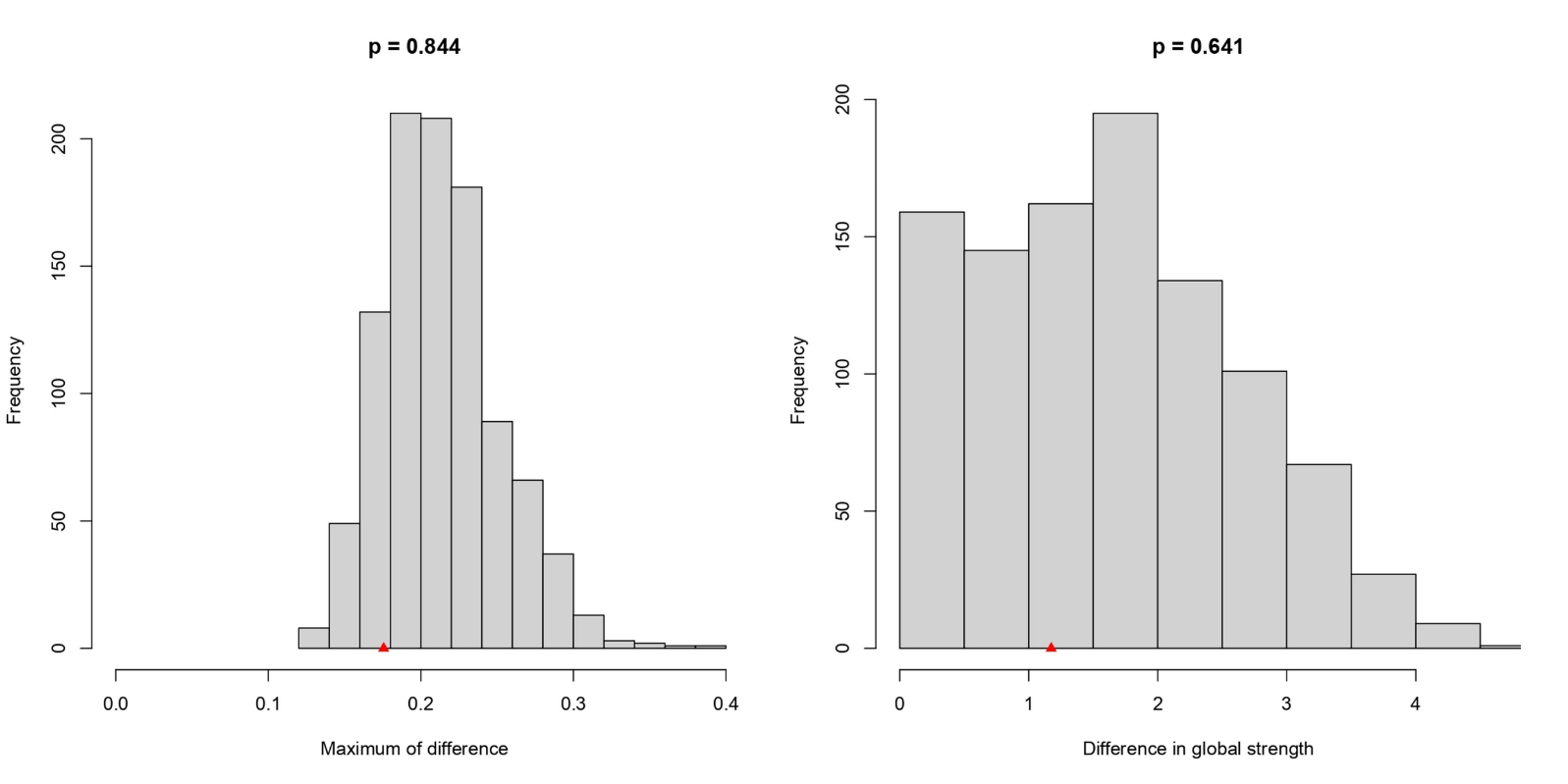


(f) Network invariance test and global strength test between employed people (N=253) and unemployed people (N=123).


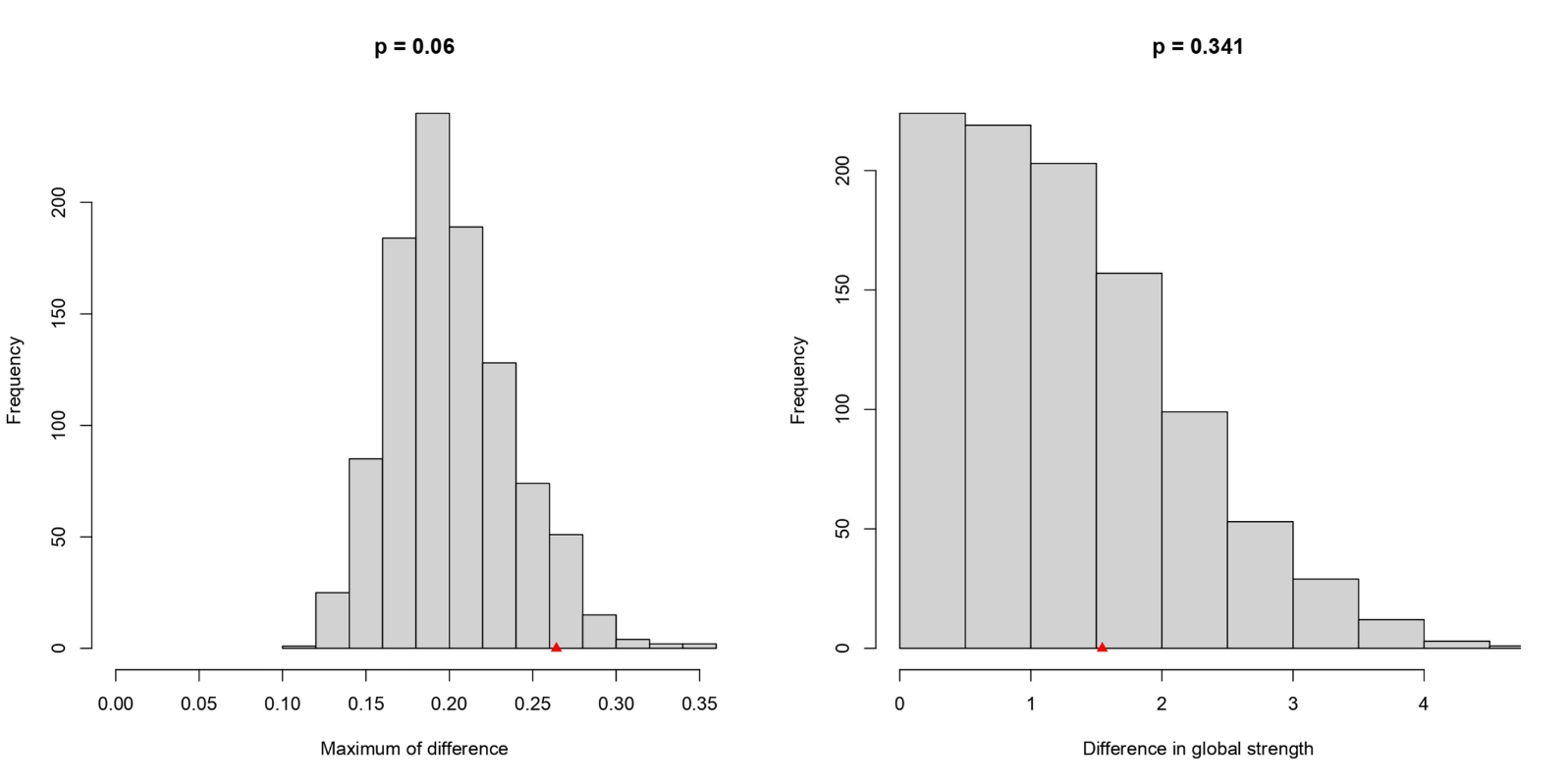


(g) Network invariance test and global strength test between people live in Hong Kong Island & Kowloon (N=144) and people live in New Territories (N=232).

**Fig S5.** **Estimated networks for subgroups based on (a) age, (b) gender, (c) education, (d) income, (e) marital status, (f) employment, and (e) residential location.** In the network, nodes represent items and edges represent associations between items. Nodes in different colors represent different categories: orange for anxiety, green for depression, yellow for perception of COVID-19, purple for perception of neighborhood, and light blue for daily activities. Thicker solid and deeper blue edges mean higher positive associations, while thicker dashes and deeper orange edges represent higher negative associations.


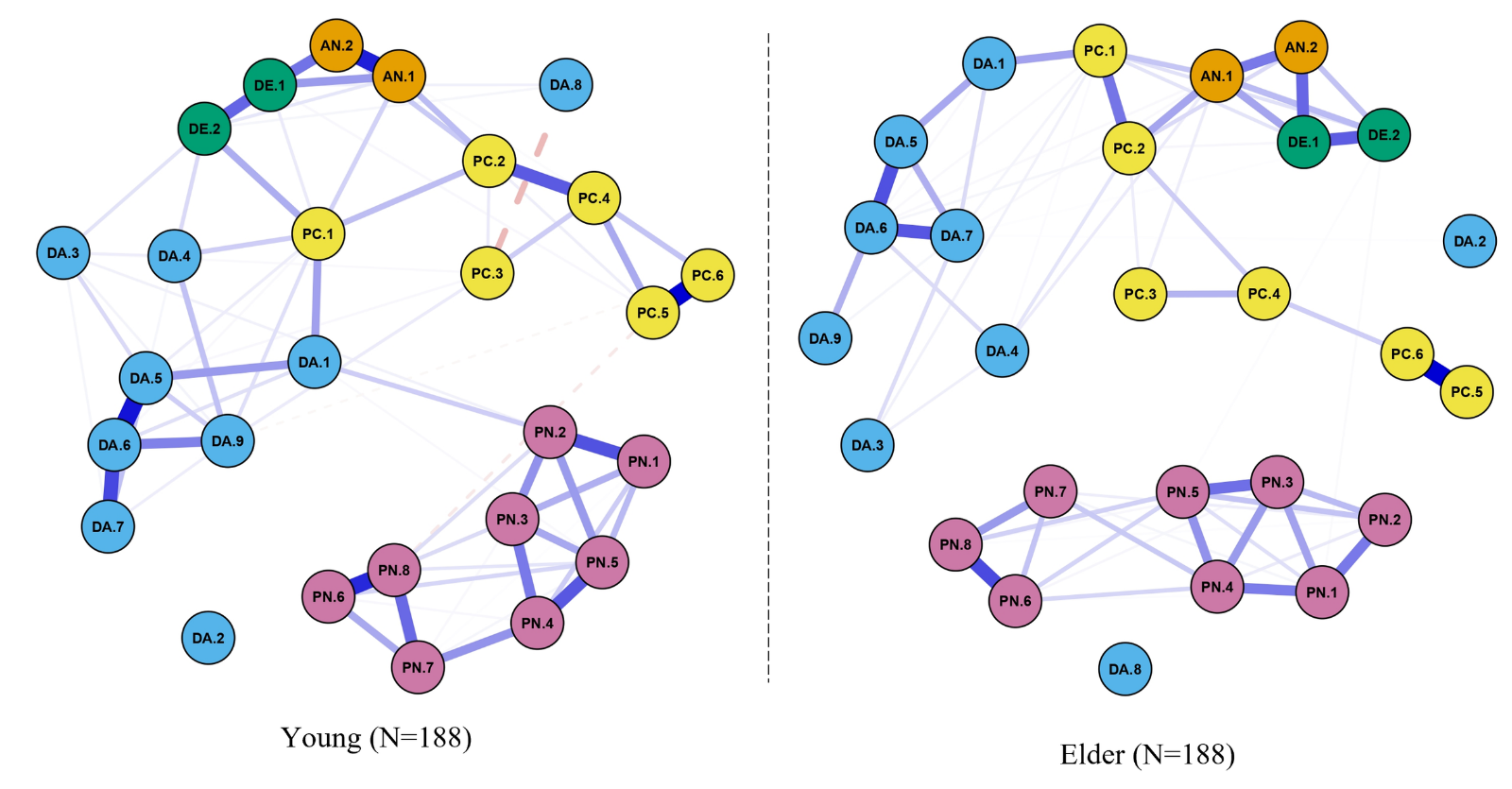


(a) Estimated networks for young people (N=188) and elder people (N=188).


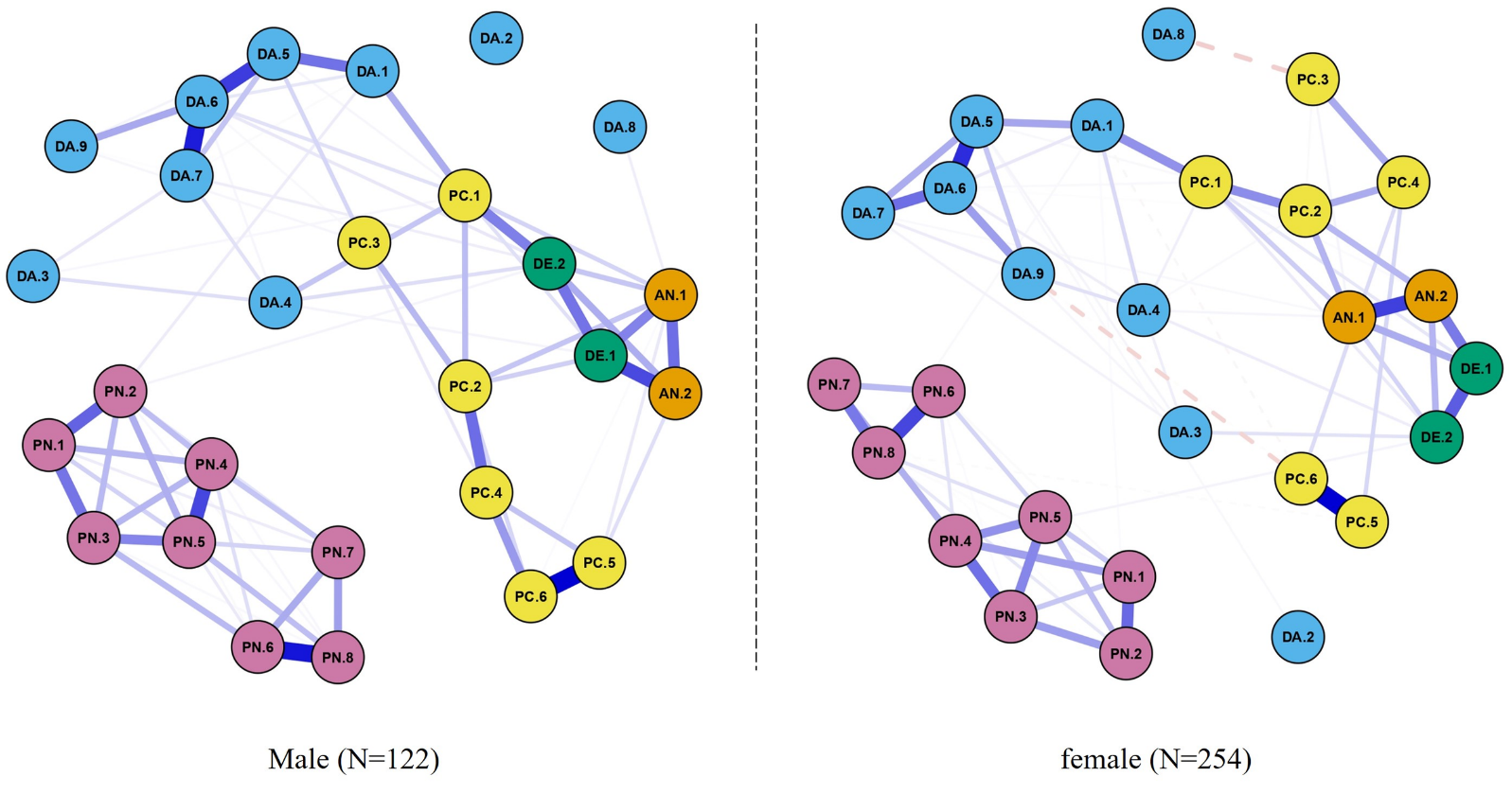


(b) Estimated networks for males (N=122) and females (N=254).


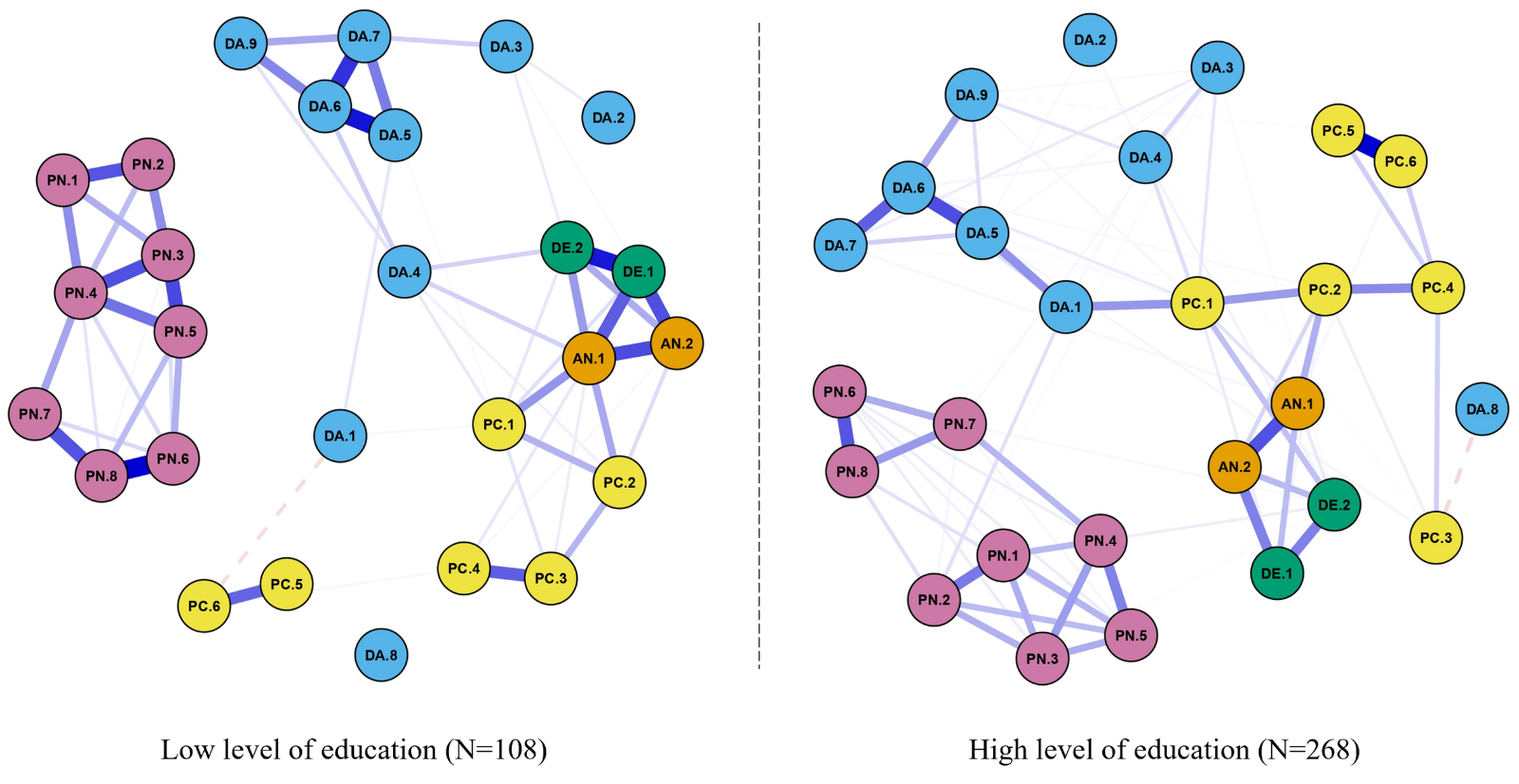


(c) Estimated networks for people with low level of education (N=108) and people with high level of education (N=268).


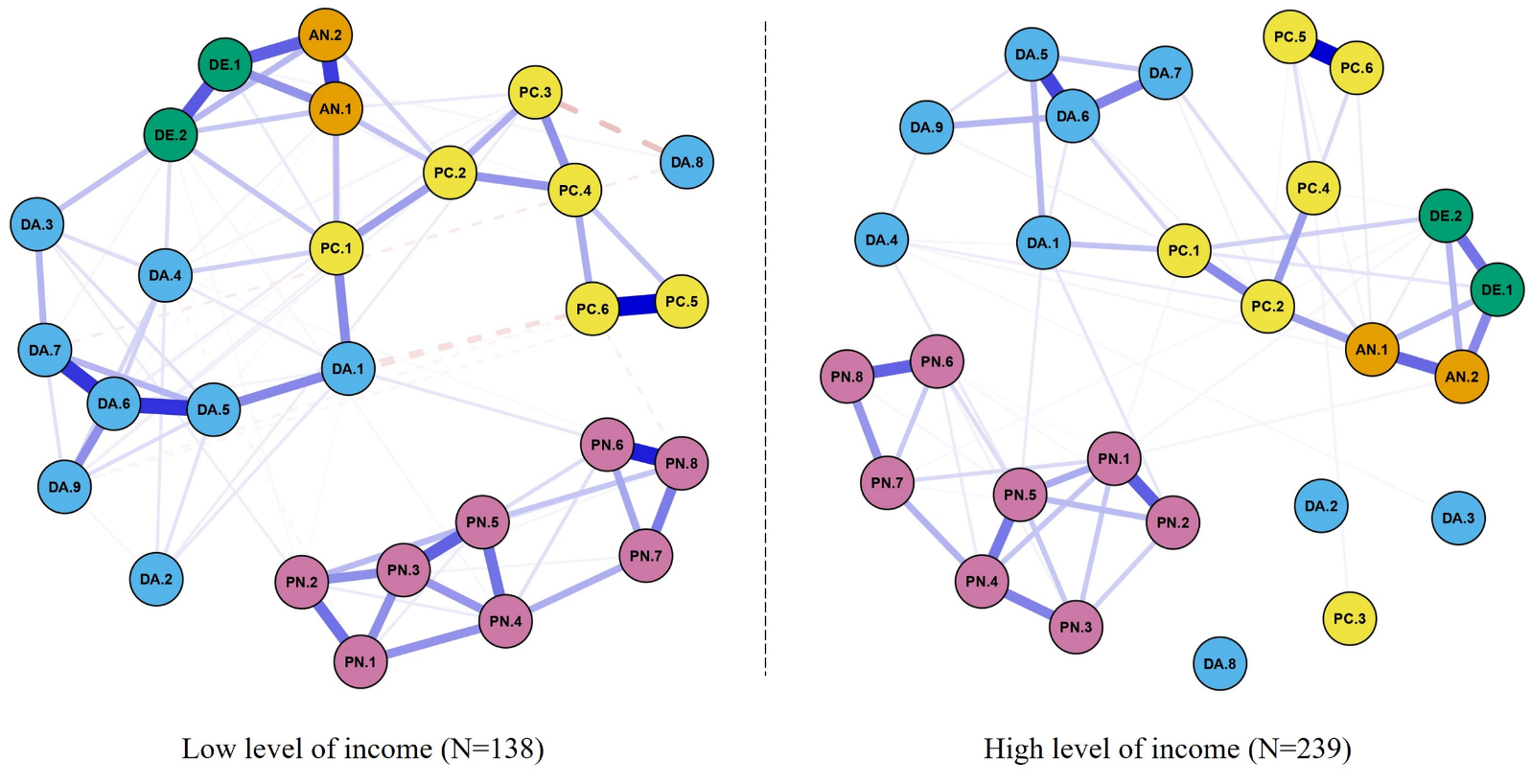


(d) Estimated networks for between people with low level of income (N=138) and people with high level of income (N=238).


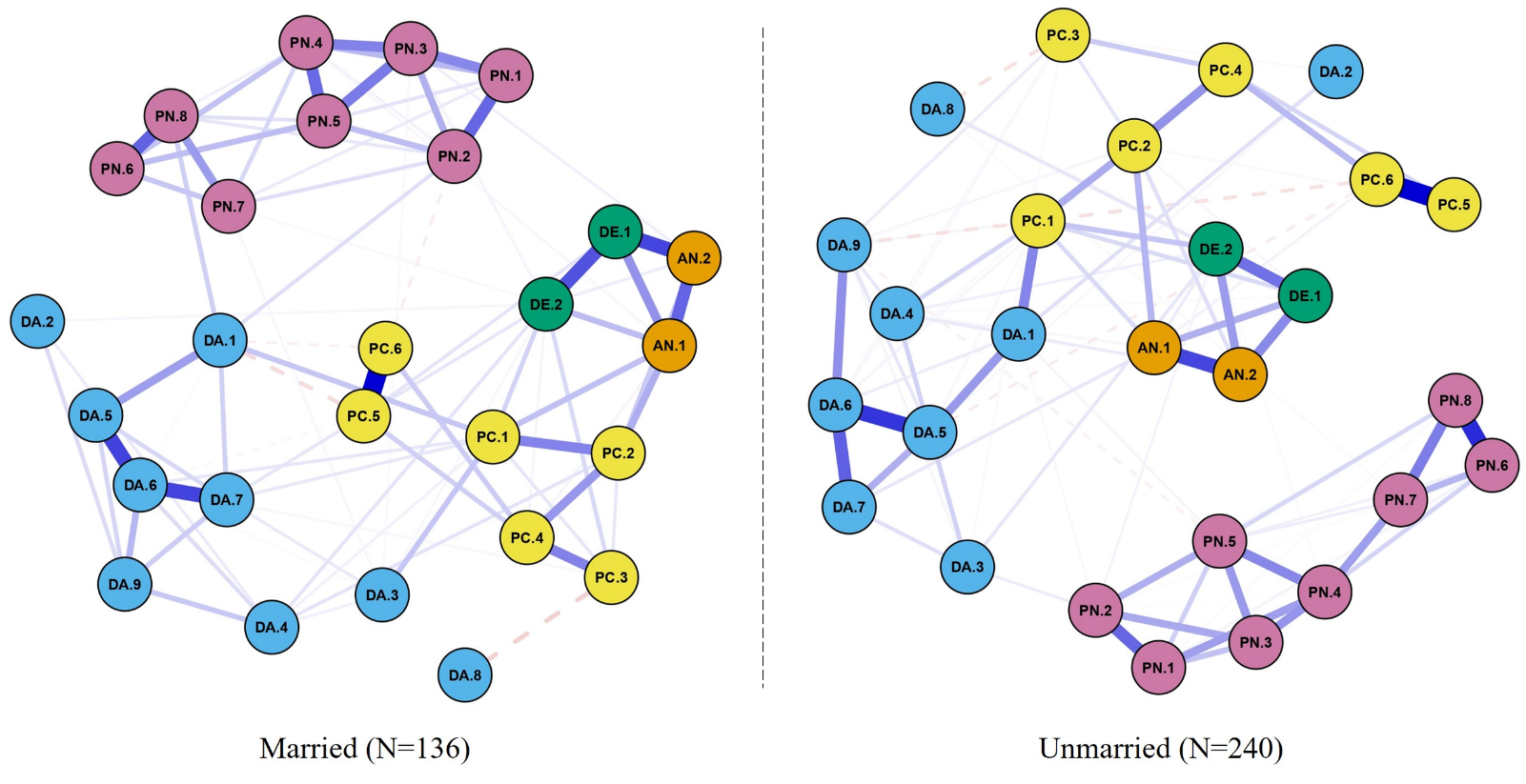


(e) Estimated networks for married (N=136) and unmarried (N=240).


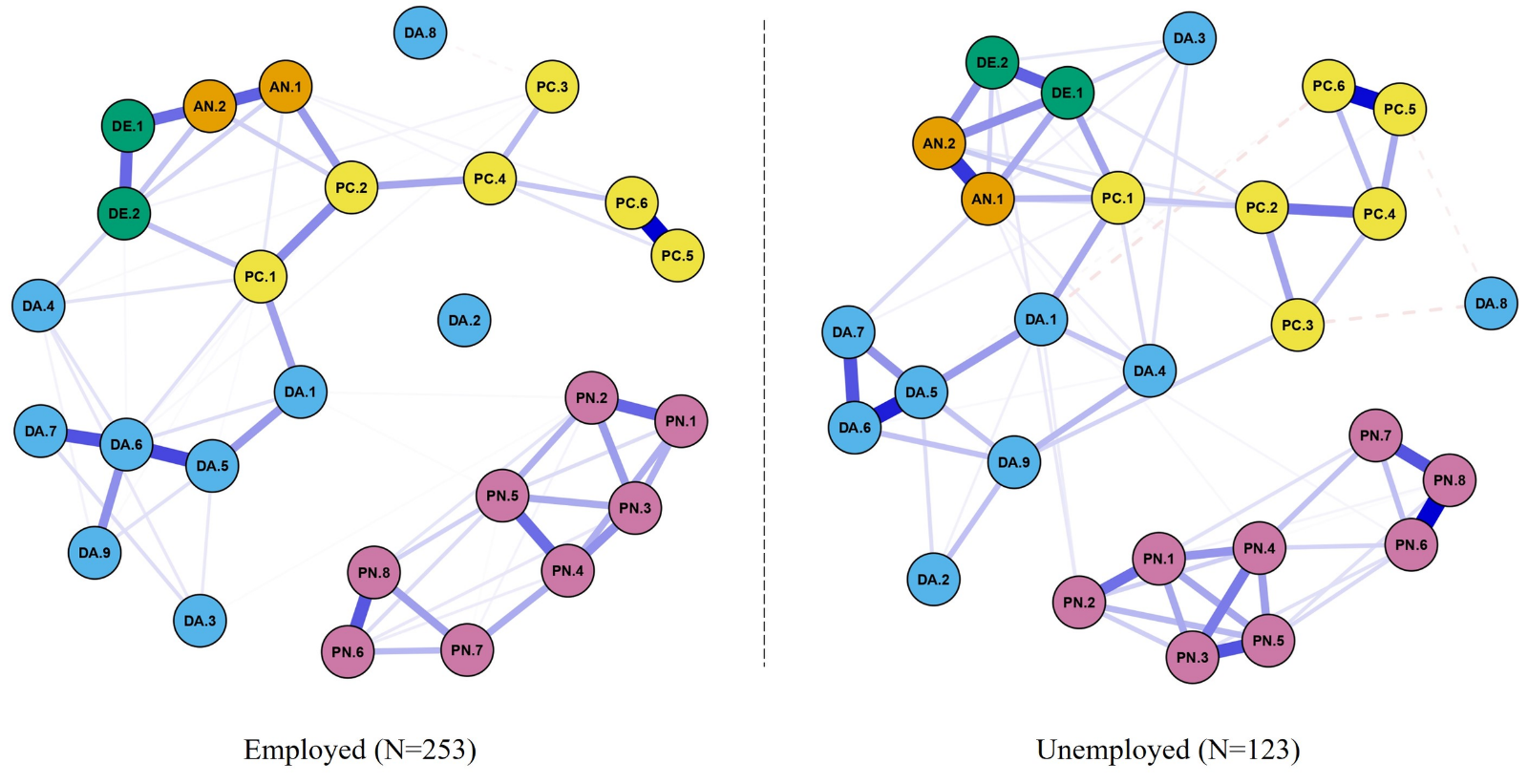


(f) Estimated networks for employed people (N=253) and unemployed people (N=123).


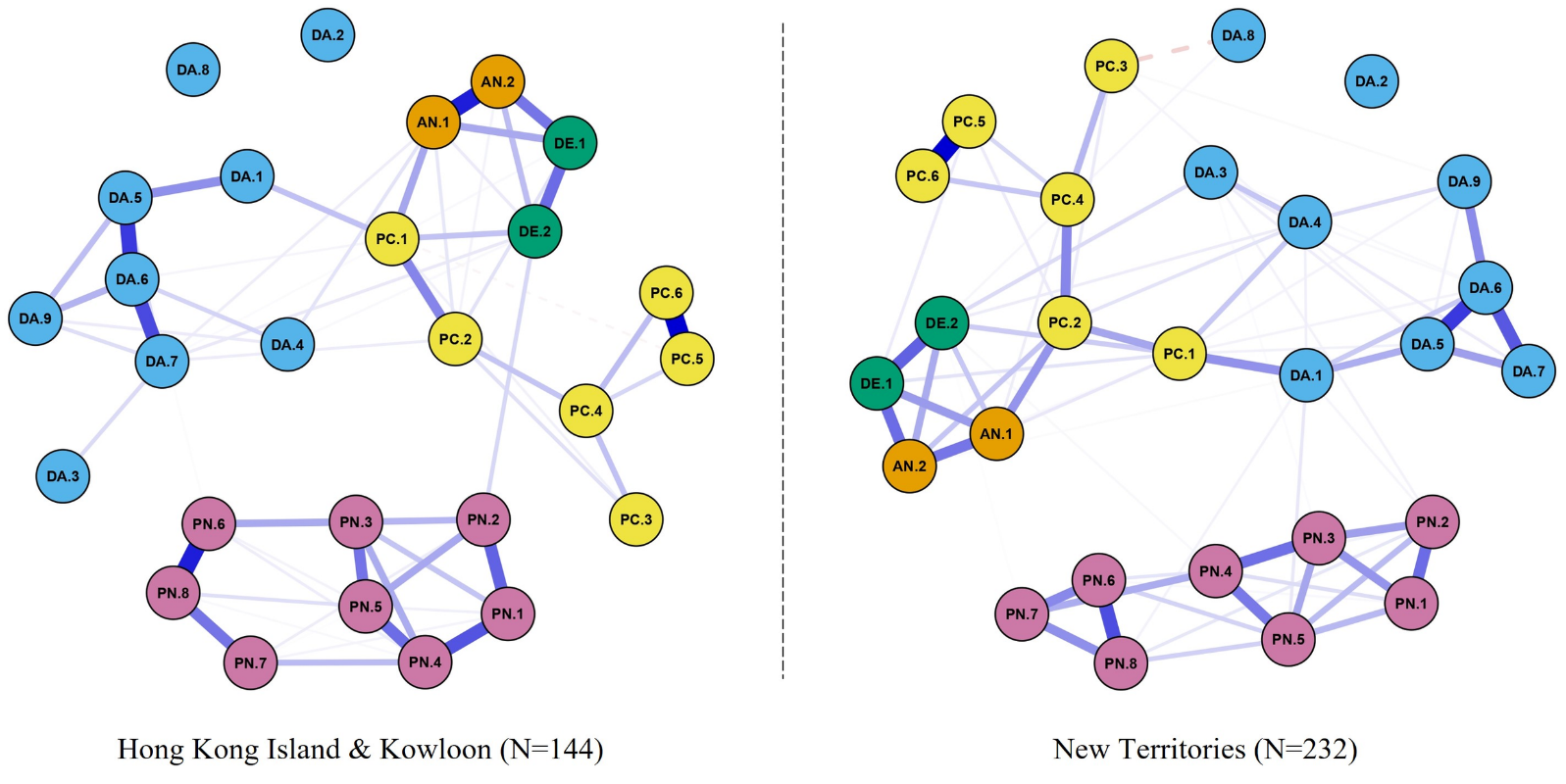


(g) Estimated networks for people live in Hong Kong Island & Kowloon (N=144) and people live in New Territories (N=232).

**References**

1. Epskamp S, Borsboom D, Fried EI. Estimating psychological networks and their accuracy: A tutorial paper. Behavior Research Methods. 2018;50(1):195-212.

2. Efron B. Bootstrap Methods: Another Look at the Jackknife. In: Kotz S, Johnson NL, editors. Breakthroughs in Statistics: Methodology and Distribution. New York, NY: Springer New York; 1992. p. 569-93.

3. Zhao Y-J, Zhang S-F, Li W, Zhang L, Guo T, Cheung T, et al. Associations between depressive symptoms and quality of life among residents of Wuhan, China during the later stage of the COVID-19 pandemic: A network analysis. Journal of affective disorders. 2022.

4. Cohen J. Statistical power analysis for the behavioral sciences: Routledge; 2013.

5. Chernick MR. Bootstrap methods: A guide for practitioners and researchers: John Wiley & Sons; 2011.

6. Saha S. Improving literacy as a means to reducing health disparities. Journal of general internal medicine. 2006;21(8):893.

7. Daly M, Sutin AR, Robinson E. Longitudinal changes in mental health and the COVID-19 pandemic: Evidence from the UK Household Longitudinal Study. Psychological medicine. 2022;52(13):2549-58.

8. Sutin AR, Robinson E, Daly M, Gerend MA, Stephan Y, Luchetti M, et al. BMI, weight discrimination, and psychological, behavioral, and interpersonal responses to the coronavirus pandemic. Obesity. 2020;28(9):1590-4.
